# Whole-genome resequencing reveals genomic variation and dynamics in Ethiopian indigenous goats

**DOI:** 10.3389/fgene.2024.1353026

**Published:** 2024-05-24

**Authors:** Oumer Sheriff, Abulgasim M. Ahbara, Aynalem Haile, Kefyalew Alemayehu, Jian-Lin Han, Joram M. Mwacharo

**Affiliations:** ^1^ Department of Animal Science, Assosa University, Assosa, Ethiopia; ^2^ Department of Animal Production and Technology, Bahir Dar University, Bahir Dar, Ethiopia; ^3^ Biotechnology Research Institute, Bahir Dar University, Bahir Dar, Ethiopia; ^4^ Department of Zoology, Faculty of Sciences, Misurata University, Misurata, Libya; ^5^ Animal and Veterinary Sciences Scotland's Rural College (SRUC) and The Centre for Tropical Livestock Genetics and Health (CTLGH), The Roslin Institute Building, Edinburgh, United Kingdom; ^6^ Resilient Agricultural Livelihood Systems Program (RALSP), International Center for Agricultural Research in the Dry Areas (ICARDA), Addis Ababa, Ethiopia; ^7^ Ethiopian Agricultural Transformation Institute, Amhara Agricultural Transformation Center, Bahir Dar, Ethiopia; ^8^ CAAS-ILRI Joint Laboratory on Livestock and Forage Genetic Resources, Institute of Animal Science, Chinese Academy of Agricultural Sciences, Beijing, China; ^9^ Livestock Genetics Program, International Livestock Research Institute, Nairobi, Kenya

**Keywords:** Africa, *Capra hircus*, genome dynamics, pooled heterozygosity, population differentiation, whole genome

## Abstract

Ethiopia has about 52 million indigenous goats with marked phenotypic variability, which is the outcome of natural and artificial selection. Here, we obtained whole-genome sequence data of three Ethiopian indigenous goat populations (Arab, Fellata, and Oromo) from northwestern Ethiopia and analyzed their genome-wide genetic diversity, population structure, and signatures of selection. We included genotype data from four other Ethiopian goat populations (Abergelle, Keffa, Gumuz, and Woyto-Guji) and goats from Asia; Europe; and eastern, southern, western, and northern Africa to investigate the genetic predisposition of the three Ethiopian populations and performed comparative genomic analysis. Genetic diversity analysis showed that Fellata goats exhibited the lowest heterozygosity values (Ho = 0.288 ± 0.005 and He = 0.334 ± 0.0001). The highest values were observed in Arab goats (Ho = 0.310 ± 0.010 and He = 0.347 ± 4.35e−05). A higher inbreeding coefficient (F_ROH_ = 0.137 ± 0.016) was recorded for Fellata goats than the 0.105 ± 0.030 recorded for Arab and the 0.112 ± 0.034 recorded for Oromo goats. This indicates that the Fellata goat population should be prioritized in future conservation activities. The three goat populations showed the majority (∼63%) of runs of homozygosity in the shorter (100–150 Kb) length category, illustrating ancient inbreeding and/or small founder effects. Population relationship and structure analysis separated the Ethiopian indigenous goats into two distinct genetic clusters lacking phylogeographic structure. Arab, Fellata, Oromo, Abergelle, and Keffa represented one genetic cluster. Gumuz and Woyto-Guji formed a separate cluster and shared a common genetic background with the Kenyan Boran goat. Genome-wide selection signature analysis identified nine strongest regions spanning 163 genes influencing adaptation to arid and semi-arid environments (*HOXC12, HOXC13, HOXC4, HOXC6*, and *HOXC9*, *MAPK8IP2*), immune response (*IL18, TYK2, ICAM3, ADGRG1,* and *ADGRG3*), and production and reproduction (*RARG* and *DNMT1*). Our results provide insights into a thorough understanding of genetic architecture underlying selection signatures in Ethiopian indigenous goats in a semi-arid tropical environment and deliver valuable information for goat genetic improvement, conservation strategy, genome-wide association study, and marker-assisted breeding.

## Introduction

Goats (*Capra hircus*) are of economic, nutritional, and cultural significance to humankind. Their domestication is believed to have happened in the Near East around 11,000 years ago from a mosaic of wild bezoar populations (*Capra aegagrus*) (Zeder, 2008; Daly et al., 2018). Human migrations and trade dispersed goats to diverse environments, and through adaptation, they integrated successfully into these environments.

The detection of genomic regions under positive selection can give insights into phenotypic evolution driven by different breeding objectives or adaptation to local environments (Andersson and Georges, 2004). Over the last decade, many genome-wide selection signatures have been detected in different goat breeds that are associated with production (Wang et al., 2016), liter size (Lai et al., 2016; Guang-Xin et al., 2019; Tao et al., 2021; Wang et al., 2021), adaptation (Kim et al., 2016; Wang et al., 2016), disease resistance (Lee et al., 2016), cashmere fiber (Li et al., 2017) and multiple traits (Guo et al., 2018). However, there are no such studies on the Ethiopian indigenous goats except Berihulay et al. (2019) whohowever, analyzed only two Ethiopian goat populations, Abergelle and Begait. In general, the studies have demonstrated how positive selection acting on complex traits has changed the genetic composition of domestic goats.

Ethiopia is home to ∼52 million goats (CSA, 2021), a great majority of which are indigenous genotypes. They occur in large flock sizes kept by pastoralists in arid and semi-arid environments, while small flock sizes maintained by agro-pastoralists are widely distributed in the highlands (Abegaz et al., 2014). Using morphometric traits, FARM-Africa (1996) classified Ethiopian indigenous goats into 13 populations. However, this classification may have inadvertently classified genetically similar populations as separate entities.

Among the indigenous goat populations of Ethiopia, Arab, Fellata, and Oromo are known to be well-adapted to Ethiopia’s semi-arid region of Benishangul-Gumuz (Getinet et al., 2005) and represent the primary livestock species and breeds raised in the area. The region’s altitudinal landscape ranges from 550 m to 2,500 m above sea level (Sinmegn et al., 2014), and its rich ancient and recent human socio-economic, political, and cultural history could have impacted the genome landscape of the indigenous goats. For instance, the two main ethnicities in Benishangul-Gumuz (Berta/Arab and Gumuz) were historically closely associated with neighboring areas of Sudan (where they extend) and, to a lesser extent, with the Ethiopian highlands. Various trade routes dating to the Axumite era (100–940 AD) met in Benishangul-Gumuz, where goods, including gold, livestock, iron, coffee, ivory, and honey, were exchanged. The Axumite kingdom was a trading empire with its hub in Eritrea and northern Ethiopia, which at times extended across most of present-day Eritrea, northern Ethiopia, Western Yemen, and parts of eastern Sudan (Fattovich, 2019). Furthermore, between 1979 and the mid-1980s, there was a relocation of a large population of inhabitants from the Ethiopian highlands (Amhara) to Benishangul-Gumuz due to drought and famine (Mulatu, 1991; Gebru, 2009). The region is also known for its intense solar radiation, feed and water scarcity, and tsetse infestation (Duguma et al., 2015). We, therefore, hypothesize that these historical, contemporary, and environmental events could have impacted the genetic makeup of indigenous goats from Benishangul-Gumuz, which could have contributed to their genetic differentiation, admixture with other Ethiopian indigenous goats, and adaptation to such an environment.

In this study, using whole-genome sequence data, we investigated i) the genetic diversity and population structure of three Ethiopian indigenous goat populations while mapping their genetic profiles to other goats from Africa, Asia, and Europe and ii) signatures of selection associated with adaptation to arid and semi-arid environments, immune response, and production and reproduction. For the latter, we present an assessment of the genome structure between Arab, Fellata, and Oromo goats descended from a semi-arid tropical environment in Ethiopia and that of Tibetan goats from a cool highland temperate environment in China and exposed to contrasting selection pressures, natural *versus* artificial, between the following population pairs: Arab vs. Tibetan, Fellata vs. Tibetan, and Oromo vs. Tibetan.

## Materials and methods

### Animals and whole-genome sequencing

Thirty (30) unrelated animals representing three northwestern Ethiopia indigenous goat populations (Arab, Fellata, and Oromo) were sampled for this study. Whole blood was collected from each individual by puncturing the jugular vein using EDTA-coated vacutainer tubes while adhering to the guidelines on animal welfare and care of the Ministry of Livestock and Fisheries of the Federal Democratic Republic of Ethiopia. Genomic DNA was extracted from whole blood using DNeasy^®^ Blood and Tissue kit (https://www.QIAGEN.com, accessed on 11 September 2019) following the manufacturer’s protocol with a few modifications. The integrity of the extracted DNA was checked in a 1% agarose gel. The concentration and purity of the DNA were determined by spectrophotometer readings at 260 nm and 280 nm, respectively (DeNovix Inc., Wilmington, DE, USA). Whole-genome sequencing at a depth of 10X was done with the NovaSeq 6000™ platform at Tianjin Noozhiyuan Technology Co., Ltd.

Genotype data from 60 animals representing ten goat populations were obtained from the VarGoats project (available from the European Nucleotide Archive (ENA), project number PRJEB37507, accessed on 16 August 2021) in FASTQ format and from which we extracted filtered genotype data. We included Ethiopian (Abergelle, Keffa, Gumuz, and Woyto-Guji) and Kenyan (Boran) goat populations to represent East African goats, Malawian (Thylo) to represent South African goats, Malian (Guera) to represent West African goats, Moroccan (Unknown) to represent North African goats, French (Saanen) to represent European goats, and Chinese (Tibetan) to represent Asian goats. A detailed description of the environmental characteristics of the geographic areas of the study populations is given in Supplementary Table S1.

### Read alignment and variant calling

Sequence read quality was evaluated with FastQC v0.11.5. Illumina adapter reads were trimmed with Trimmomatic v0.36 (Bolger et al., 2014) based on the following criteria: Slidingwindow:4:15, Leading:3, Trailing:3, and Minlen:36. The clean reads were mapped to the ARS1 *C. hircus* reference genome assembly (RefSeq number GCF_001704415.1) (Bickhart et al., 2017) using the Burrows–Wheeler Aligner (BWA-MEM algorithm v0.7.17) (Li, 2013) with default parameters except for -t 8 -M” and “-R” to add read groups. Each sequence alignment map (SAM) (Li and Durbin, 2009) was sorted and converted to a binary alignment map (BAM) (Li and Durbin, 2009) using Picard (https://broadinstitute.github.io/picard/) “SortSam” v2.22.8. The same software was also used to mark duplicate reads with “MarkDuplicates.” Each BAM file was indexed using “BuildBamIndex,” and read groups were added using “AddOrReplaceReadGroups” of Picard v2.22.8. GATK (Genome Analysis Toolkit; McKenna et al., 2010) BaseRecalibrator and ApplyBQSR v4.1.7.0 were used for base quality score recalibration. The “known-sites” file that is necessary for the BQSR step was computed for each individual. GATK HaplotypeCaller v4.1.7.0 (Poplin et al., 2018) was run to call the variants in the genomic variant call format (GVCF) mode using the GVCF parameter on the pre-processed BAM files. GATK’s CombineGVCFs v4.1.7.0 was then used to aggregate all GVCF files per scaffold. GenotypeGVCFs v4.1.7.0 of GATK was used to perform joint genotyping and output multisample raw variant call format (VCF) (Danecek et al., 2011) per chromosome/scaffold.

### Filtering process

The multisample raw VCF of each goat population was filtered by VariantRecalibrator v4.1.7.0 of GATK. Two training resources, one with true sites (“known = false, training = true, truth = true, prior = 15.0”) and the other with non-true sites (“known = true, training = false, truth = false, prior = 2.0”) used for recalibration were dbSNP variants obtained from Ensembl v105. The variant call annotations (for SNPs and InDels) DepthOfCoverage (DP), QualByDepth (QD), RMSMappingQuality (MQ), MappingQualityRankSumTest (MQRankSum), ReadPosRankSumTest (ReadPosRankSum), FisherStrand (FS), and StrandOddsRatio (SOR) were used for VariantRecalibrator. Based on the SNP tranches (Supplementary Figure S1), no false-positive variants were observed in the 90 tranche. Therefore, the 99 tranche was included to increase the sensitivity of variant discovery. We considered the highest tranche (99.9–100) as a false positive and excluded it. The remaining variants (SNPs and InDels) were then recalibrated at the truth sensitivity filter level (tranche) of 99 using ApplyVQSR v4.1.7.0 of GATK. In general, the entire filtration process resulted in a high confidence set of 35,161,094 biallelic autosomal SNPs and 3,737,445 InDels. The final set of SNPs was annotated using variant effect predictor (VEP) v104.3.

### Genomic diversity analyses

Genomic diversity for each population was analyzed using various metrics, including the proportion of polymorphic SNPs (*Pn*), nucleotide diversity (*π*), and genomic expected (*He*) and observed (*Ho*) heterozygosity. Estimates of *Pn*—the fraction of total SNPs that displayed both alleles—were calculated as the proportion of SNPs with minor allele frequency (MAF) greater than 0.01. The *π* analysis (Nei and Li, 1979) is a method that uses SNPs to calculate the average difference between any two nucleotide sequences in a population. The *π* values were computed based on the sliding window method within 100-kb windows with 50 kb step size along the autosomes using the VCFtools v0.1.15 (Danecek et al., 2011). The *Ho* was calculated as the proportion of total heterozygous SNPs to the total number of sites counted in each genome, based on the ARS1 *C. hircus* reference genome. The observed and expected heterozygosity was calculated using the “--het” option of PLINK v1.9 (Purcell et al., 2007) for each genome and then averaged for each population.

The average pairwise genetic distance (D) between individuals within a population was calculated in PLINK v1.9 (Purcell et al., 2007). The average proportion of alleles shared between two individuals was calculated using the “--genome” command line in PLINK v1.9 as: $D_{ST}={IBS}_{2}{+(0.5\;X\;IBS}_{\left.1\right)}/N$ , where IBS_1_ and IBS_2_ represent the number of loci that share either one or two alleles that are identical by state (IBS) in pairwise comparisons between individuals, respectively, and N is the number of loci tested. The genetic distance between all pairwise combinations of individuals was calculated as D = 1 − $D_{\text{ST}}$ .

The runs of homozygosity (ROHs) were estimated with PLINK v1.9 by invoking the “--homozyg” option. ROHs are uninterrupted stretches of homozygous genotypes common among individuals within a population (McQuillan et al., 2008; Marras et al., 2015). The degree of ROH variation between populations was characterized based on differences in the length and number of ROH fragments between populations. The following PLINK parameters and thresholds (Purcell et al., 2007) were applied to define an ROH region: (i) minimum number of SNPs in ROH or in sliding window = 50, (ii) minimum length of ROH = 100 kb, (iii) minimum number of missing SNP in the ROH = 1, (iv) minimum allowed density of SNPs within a run = 1 SNP/100 kb, (v) minimum number of heterozygous SNPs in each ROH = 1, and (vi) maximum gap between consecutive homozygous SNPs = 1 Mb. ROHs enable reliable estimation of the level of inbreeding. We estimated the ROH-derived genomic inbreeding coefficient ( $F_{\text{roh}}$ ) following McQuillan et al. (2008) as $F_{roh}=\sum L_{roh}/L_{auto}$ , where $L_{roh}$ is the total length of ROH of each individual in the genome, and $L_{auto}$ is the length of the goat autosomal genome (∼2400 Mb) (Kumar et al., 2018). To compare the ROH length between populations, four length categories were allocated: 100–150 Kb, >150–250 Kb, >250–400 Kb, and >400 Kb (classified as ROH_100–150Kb_, ROH_150–250Kb_, ROH_250–400Kb_, and ROH_>400Kb,_ respectively). For each ROH length category, we summed all ROH per animal and averaged this per population. In order to investigate the potential of our approach, we focused on two length classes: from 100 kb to 400 kb to investigate ancient events and >400 kb to address recent events.

### Population genetic structure analyses

We used three methods to detect the genetic differentiation and structure among the studied goat populations: a principal component analysis (PCA) to visualize patterns in relationships between individuals using the “--pca” command line in PLINK v1.9 (Purcell et al., 2007); Treemix v.1.13 (Pickrell and Pritchard, 2012) to construct a maximum likelihood (ML) tree and visualize population splits and the directionality of gene flows between populations; and ADMIXTURE v.1.3 (Alexander et al., 2009) to perform the ancestry proportion of individual genomes.

The PCA was performed at three levels: global, East African, and Ethiopian. To summarize the relationship among individuals, we plotted the first two eigenvectors. For this analysis, we filtered out SNPs with minor allele frequency (MAF) < 0.01, leaving 27,728,833 SNPs that were used. The graphical display of PCA results (PC1 and PC2) was visualized with GENESIS (Buchmann and Hazelhurst, 2014).

In the TreeMix analysis, the number of migration events (m) varied between one (1) (migration between two populations) and thirteen (13) (migration between all populations). The TreeMix plotting script was used to visualize the trees and the proportion and direction of gene flow events between the 13 goat populations with R. The number of gene flow events that best fit the data was identified using the fraction of the variance in the sample covariance matrix explained by the model covariance matrix (Pickrell and Pritchard, 2012). To evaluate the confidence of the trees’ edges and nodes, 1000 bootstrap replicates were performed. Before running migration events, it is important to define the position of the root using prior information about known out-groups (Pickrell and Pritchard, 2012). We used the Tibetan goat population as the root (-root) in our analyses. Furthermore, to test for admixture among breeds and to assess the statistical significance of gene flow events, the THREEPOP and FOURPOP functions (implemented in TreeMix) were run to calculate the *f3* and *f4* statistics, respectively (Ahbara et al., 2019). For the *f3* test (A; B, C), the putative admixture of a target population (A) is tested against two source populations (B, C). A significantly negative value of the resulting Z-score indicates A is admixed. The *f4* test (A, B; C, D) investigates the tree topology of four populations. A significantly positive Z-score suggests gene flow between populations A and C or B and D that surpasses any gene flow between populations A and D or B and C. A significantly negative Z-score, however, indicates gene flow between populations A and D or B and C that surpasses any gene flow between A and C or B and D. Standard errors were estimated using blocks of 500 SNPs.

For the ADMIXTURE analysis, we used 8,344,942 autosomal SNPs that were retained after we pruned the 27,728,833 SNPs used in PCA for linkage disequilibrium (LD). ADMIXTURE was run for *K* = 2 to *K* = 13. A five-fold cross-validation (CV) procedure was applied to determine the optimal number of clusters (K). GENESIS (Buchmann and Hazelhurst, 2014) was used to display the ADMIXTURE OUPUTS.

### Genomic selection signature analysis

We implemented two methods for the selection signature analysis: i) genetic differentiation based on *F*_*ST*_ (Weir and Cockerham, 1984) and ii) within-group pooled heterozygosity (*H*_*P*_) (Rubin et al., 2010). A sliding window approach was used to perform the *F*_*ST*_ analyses with VCFtools (v.0.1.15) using a 100-kb sliding window size (≥30 SNPs) and a 25-kb step size according to the method used previously (Lai et al., 2016; Guo et al., 2018) following the equations: *F*_*ST*_ *=* $1-\;\left(p1q1+p2q2\right)/2prqr$ ; where p1, p2 and q1, and q2 are the frequencies of alleles A and a in the first and second group of the test populations, respectively, and pr and qr are the frequencies of alleles A and a, respectively, across the tested groups (Zhi et al., 2018). The *F*_*ST*_ values were standardized into Z-scores as follows: $Z\left(F_{ST}\right)=Z\left(Fst\;–\;µ\left(F_{ST}\right)\right)/\sigma \left(F_{ST}\right);$ where µ is the mean, and σ is the standard deviation of *F*_*ST*_ derived from all the windows tested between test groups. Finally, the top 1% of windows showing both extremely high *F*_*ST*_ values (the top 1% of Z (*F*_*ST*_) distributions) and extremely low *H*_*P*_ scores (the bottom 1% of *ZH*_*P*_ distributions) (Guo et al., 2018) were proposed to be under selection.

The *H*_*P*_ values were also estimated using a window size of 100 kb and a sliding step size of 25 kb (Guo et al., 2018) with the formula: *H*_*P*_ *=*  2∑nMaj∑nMin/∑nMaj+∑nMin^2 ; Where *∑nMaj* and *∑nMin* are the sums of major and minor allele frequencies, respectively, for all the SNPs in the 100-kb window. The values for the *H*_*P*_ calculated for each window size were then Z-transformed using the equation: Z (HP) = $\left(Hp\;–\;µ\left(Hp\right)\right)/\left(\sigma \left(Hp\right)\right),$ where µ is the mean and σ is the standard deviation of *H*_*P*_. Because sex chromosomes and autosomes are subjected to different selective pressures and have different effective population sizes, we calculate the *ZHp* for autosomes of specific breeds only. Putative genomic regions that overlapped between these two approaches were defined as candidate selective regions.

### Candidate gene analysis

Based on genome annotation, a gene was deemed to show evidence of being under selection if it overlapped with an outlier genomic window based on both *ZF*_*ST*_ and *ZHp.* Hence, we annotated the candidate genomic regions using the *Ensemble BioMart* tool (https://www.ensembl.org/biomart, accessed on 16 February 2024) based on the Caprine reference genome (*ARS1*). Using *C. hircus* as a background species, we performed functional enrichment analysis of all the annotated genes using Database for Annotation, Visualization, and Integrated Discovery (DAVID, https://david.ncifcrf.gov/content.jsp?file=release.html, accessed on 16 February 2024) v6.8 (Huang et al., 2009). Categories with the threshold of adjusted *p*-value <0.05 after the Bonferroni correction were defined as significantly enriched terms and pathways. To infer gene functions, we consulted the NCBI database (https://www.ncbi.nlm.nih.gov) and reviewed the literature.

## Results

### Genome sequence mapping and SNP calling

The SNP and InDel summary statistics of the sequence parameters for each population are shown in Supplementary Tables S2, S3, respectively. The highest number of SNPs (18,266,925) and InDels (2,033,758) were detected in the Unknown (UNK) population from Morocco, while the lowest number of SNPs (14,389,837) and InDels (1,739,360) were identified in Saanen (SAN; Europe) breed and Gumuz (GUM; Ethiopia) breed, respectively. The average number of SNPs per sample ranged from 6,591,579 (Keffa (KEF); Ethiopian) to 8,866,137 (Woyto-Guji (WGU); Ethiopian). The average number of InDels ranged from 660,074 (Keffa; KEF) to 976,619 (Abergelle (ABR); Ethiopian). The heterozygosity to homozygosity (het/hom) ratio ranged from 1.03 (Keffa (KEF)) to 1.62 (Unknown (UNK)). A comparison with the *C. hircus* dbSNP database revealed that 29%–35% of the SNPs and 78%–82% of the InDels were not present in the database.

### Genomic diversity

Genomic diversity metrics (mean ± standard deviation (SD); Table 1) for each population were assessed by estimating the proportion of polymorphic SNPs (Pn), observed (Ho) and expected (He) heterozygosity, ROH-based inbreeding ( $F_{roh}$ ); nucleotide diversity (π), and average pairwise genetic distance between individuals within populations/breeds (D). The Pn ranged between 90.5% and 96%. The highest Pn was in the unknown breed (Morocco) and the lowest in Thylo (Malawi). Keffa goats had the lowest Ho (0.254 ± 0.115), π (0.0017 ± 0.001) and D_ST_ (0.264 ± 0.001) but the highest $F_{roh}$ (0.261 ± 0.326). On the other hand, Woyto-Guji had the highest Ho (0.335 ± 0.009) and π (0.0023 ± 0.001) but the lowest $F_{roh}$ (0.016 ± 0.027). The value of He was always greater than that of Ho in each breed.

**TABLE 1:** Estimates of genetic diversity parameters for each of the 13 populations analyzed in this study.

Population	N	Pn	Ho	He	F_ROH_	π	D
			Mean ± SD	Mean ± SD	Mean ± SD	Mean ± SD	Mean ± SD
Arab	10	0.928	0.310 ± 0.010	0.347 ± 4.35e−05	0.105 ± 0.030	0.0020 ± 0.01	0.280 ± 0.033
Fellata	10	0.944	0.288 ± 0.005	0.334 ± 0.0001	0.137 ± 0.016	0.0021 ± 0.001	0.281 ± 0.005
Oromo	10	0.930	0.306 ± 0.012	0.345 ± 0.0002	0.112 ± 0.034	0.0020 ± 0.001	0.281 ± 0.016
Abergelle	6	0.933	0.299 ± 0.074	0.340 ± 0.003	0.121 ± 0.210	0.0019 ± 0.001	0.280 ± 0.004
Keffa	6	0.924	0.254 ± 0.115	0.342 ± 0.004	0.261 ± 0.326	0.0017 ± 0.001	0.264 ± 0.001
Gumuz	6	0.932	0.334 ± 0.025	0.345 ± 8.80e−05	0.032 ± 0.072	0.0022 ± 0.001	0.269 ± 0.005
Woyto-Guji	6	0.939	0.335 ± 0.009	0.339 ± 9.06e−05	0.016 ± 0.027	0.0023 ± 0.001	0.296 ± 0.017
Boran	6	0.941	0.330 ± 0.011	0.337 ± 0.0002	0.020 ± 0.032	0.0022 ± 0.001	0.265 ± 0.002
Unknown	6	0.960	0.304 ± 0.023	0.328 ± 5.15e−05	0.072 ± 0.071	0.0022 ± 0.001	0.272 ± 0.007
Thyolo	6	0.905	0.332 ± 0.048	0.357 ± 7.30e−05	0.068 ± 0.135	0.0019 ± 0.001	0.274 ± 0.009
Guera	6	0.934	0.322 ± 0.029	0.346 ± 0.0007	0.069 ± 0.084	0.0020 ± 0.001	0.278 ± 0.016
Saanen	6	0.915	0.331 ± 0.038	0.369 ± 0.0011	0.174 ± 0.101	0.0018 ± 0.001	0.306 ± 0.012
Tibetan	6	0.932	0.330 ± 0.026	0.334 ± 0.0006	0.117 ± 0.077	0.0021 ± 0.001	0.275 ± 0.025

Pn, polymorphic SNPs; Ho, observed heterozygosity; He, expected heterozygosity; F_ROH_, ROH-based inbreeding; π, nucleotide diversity; D, pairwise genetic distance.

### Runs of homozygosity (ROHs)

**FIGURE 1:**
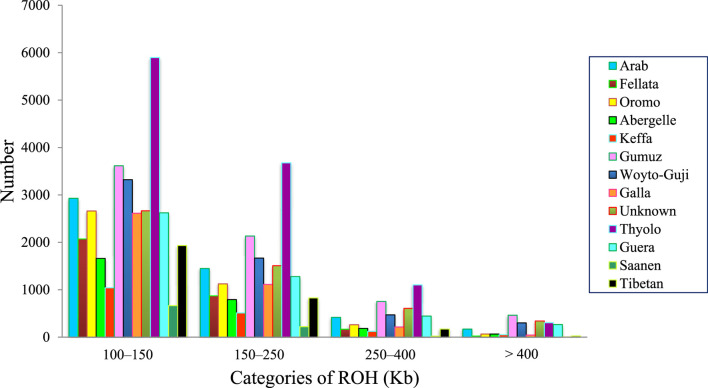
Number of ROHs for the four genome length categories (ROH_100–150Kb_, ROH_150–250Kb_, ROH_250–400Kb_, and ROH_>400Kb_) of the study goat populations.

The frequency of ROHs was calculated from homozygous sequences across each genome for the four genome length categories (ROH_100–150Kb_, ROH_150–250Kb_, ROH_250–400Kb_, and ROH_>400Kb_) analyzed here (Figure 1). A total of 57,910 ROH segments were identified across the 78 individuals. Thyolo had the highest number (10,979) of ROH segments, while Saanen had the lowest number (909) of ROH segments (Table 2). The ROHs comprised mostly (58%) the shorter (ROH_100–150Kb_) length category, while a very small proportion (∼4%) of the ROH length category was detected in the longer ROH segment (ROH_>400Kb_).

**TABLE 2:** Statistics of ROH observed in the study goat populations under different length classes.

Population	Total number of ROH	ROH number in different ROH length categories			
		ROH_100–150Kb_	ROH_150–250Kb_	ROH_250–400Kb_	ROH_>400Kb_
Arab	4964	2929	1448	418	169
Fellata	3133	2071	871	168	23
Oromo	4113	2661	1124	262	66
Abergelle	2701	1660	791	184	66
Keffa	1721	1040	510	121	50
Gumuz	6964	3615	2133	754	462
Woyto-Guji	5761	3322	1668	470	301
Boran	3984	2614	1112	214	44
Unknown	5119	2665	1506	607	341
Thyolo	10979	5897	3676	1102	304
Guera	4608	2621	1278	443	266
Saanen	909	664	220	20	5
Tibetan	2954	1932	827	170	25
Overall	57910	33691 (58.18%)	17164 (29.64%)	4933 (8.52%)	2122 (3.66%)

### Population structure

**FIGURE 2:**
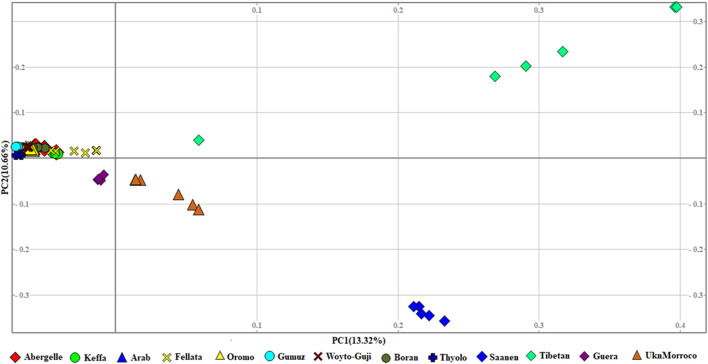
PCA plots for the first two components (PC1 and PC2, the respective variations explained in brackets) for the global goat populations.

The genetic relationship between individuals was analyzed with PCA, which we performed at three levels: global, East African, and Ethiopian. At the global level, the PCA separated the 13 populations into four groups that corresponded to their geographic origin, viz, 1) Asian, 2) European, 3) South and East African, and 4) North and West African. In this PCA, PC1 and PC2 explained 13.32% and 10.66%, respectively, of the variation in the entire genetic data (Figure 2).

**FIGURE 3:**
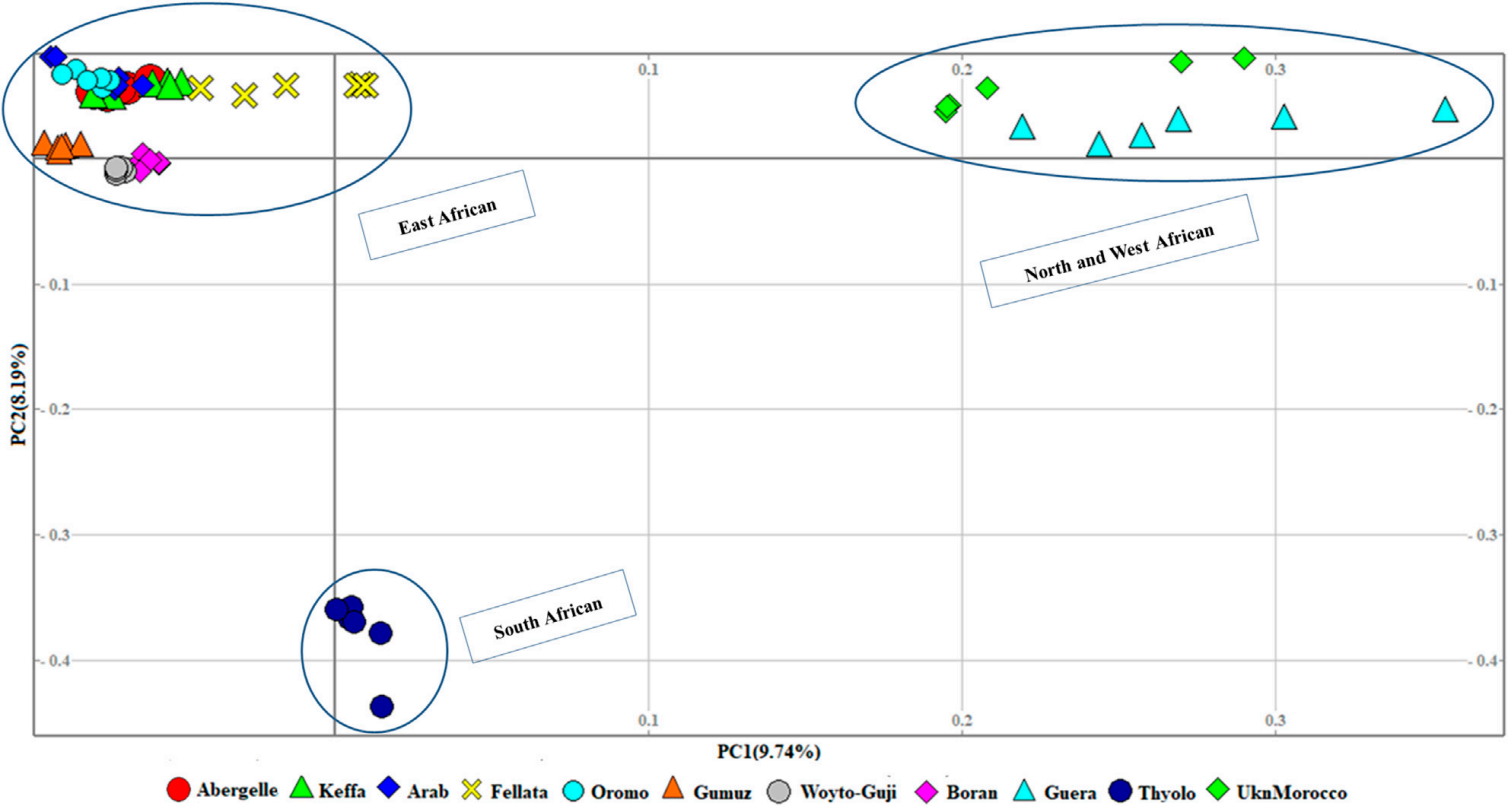
PCA plots for the first two components (PC1 and PC2, the respective variations explained in brackets) for African goats.

At the second level, which aimed to obtain a clearer picture of the genetic variation among 11 African populations (Figure 3), the PCA revealed three genetic clusters, viz, 1) East African, 2) North and West African, and 3) South African. In contrast to the global PCA, there is the separation of East African goats from the South African. In this dataset, PC1 and PC2 accounted for 17.93% of the total variation.

**FIGURE 4:**
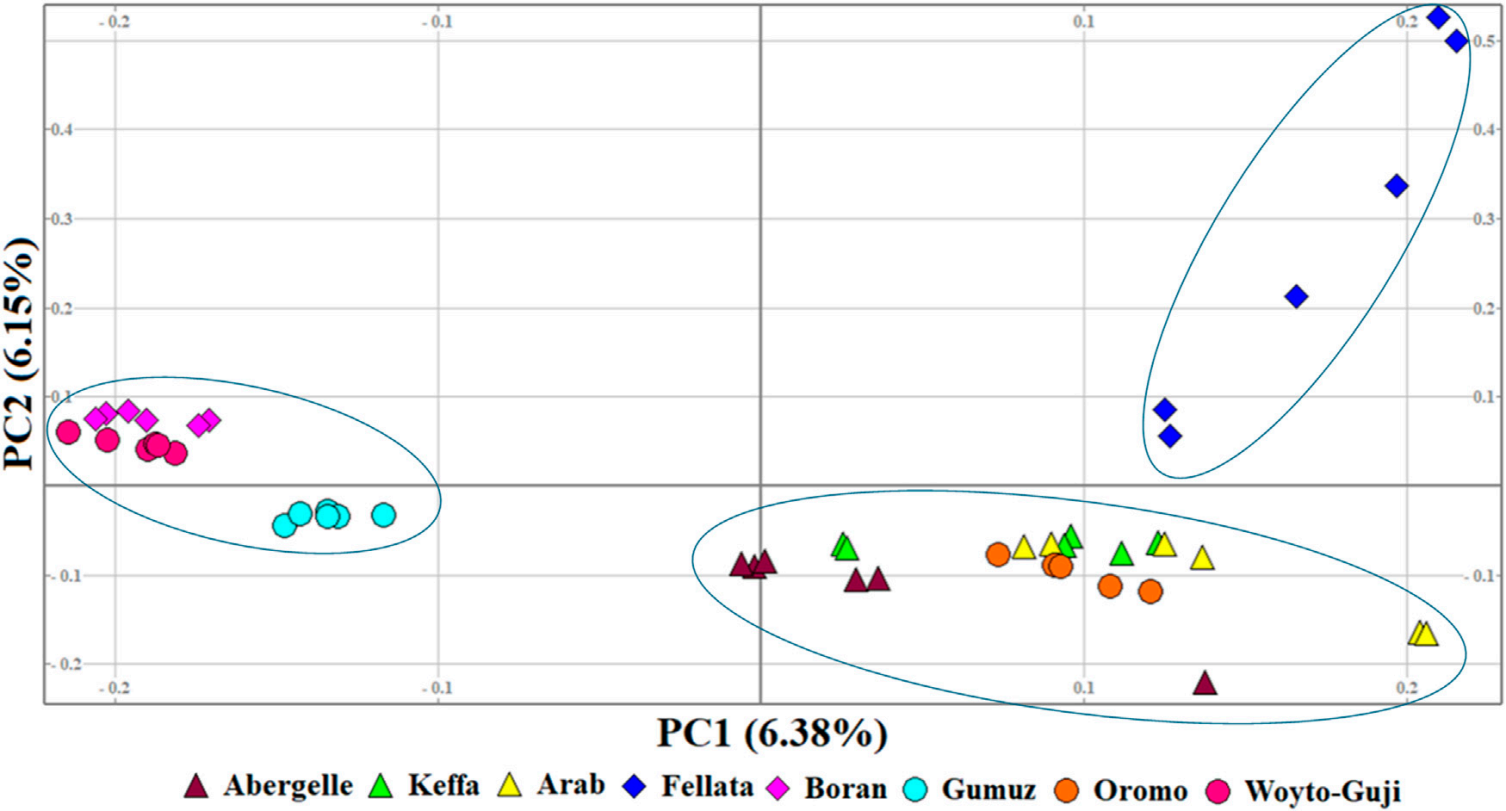
PCA plots for the first two components (PC1 and PC2, the respective variations explained in brackets) for the East African goats.

To illustrate the genetic variation among East African goats, we performed the PCA with only the eight East African goat populations (Figure 4). It revealed three genetic clusters: Cluster 1 (Boran, Gumuz, and Woyto-Guji), Cluster 2 (Abergelle, Arab, Keffa, and Oromo), and Cluster 3 (Fellata). In this dataset, PC1 and PC2 explained 12.53% of the total variation.

**FIGURE 5:**
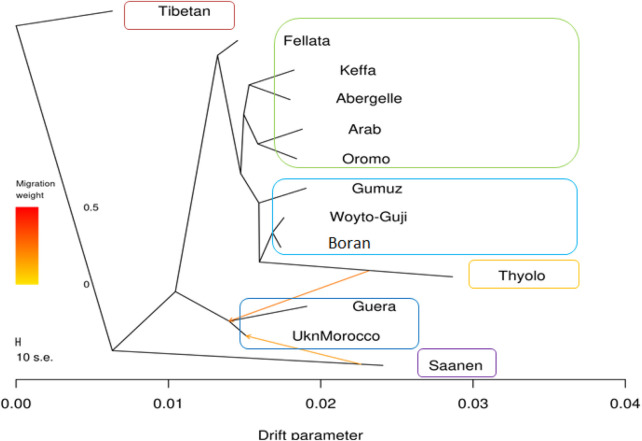
TreeMix maximum likelihood phylogenetic tree showing the relationships among the 13 goat populations. Horizontal branch lengths are proportional to the amount of genetic drift that has occurred along that branch. The scale bar on the left shows 10 times the average standard error (s.e.) of the entries in the sample covariance matrix. Two migration edges between populations are shown with arrows pointing in the direction of the recipient group and colored according to the ancestry percentage received from the donor.

The phylogenetic tree generated with TreeMix (Figure 5) revealed four genetic groups in the 13 goat populations. The groups are similar to the genotype groups seen in the PCA plot (Figure 2). As expected, it clearly distinguished Saanen and Tibetan goats. However, it clustered the South and East African goats in one group, separate from the North and West African goats, which were in another group. It also revealed two gene flow events, one from Saanen to Unknown and the other from Thyolo to Guera. Some of the populations, including Saanen, Thyolo, and Tibetan, are shown to have long branches corresponding to the amount of genetic drift.

The *f3* test failed to reveal any gene flow events between the populations analyzed (Supplementary Table S4). A complex pattern of gene flow between the study populations, which cannot be explained by a three-way model, may explain this result. The *f4* test highlighted possibilities of gene flow among various populations. The highest Z-scores (>|54|) were between Boran and Abergelle and between Unknown and Saanen (Supplementary Table S5). The lowest Z-scores (>|54|) were between Boran and Woyto-Guji and between Saanen and Abergelle.

**FIGURE 6:**
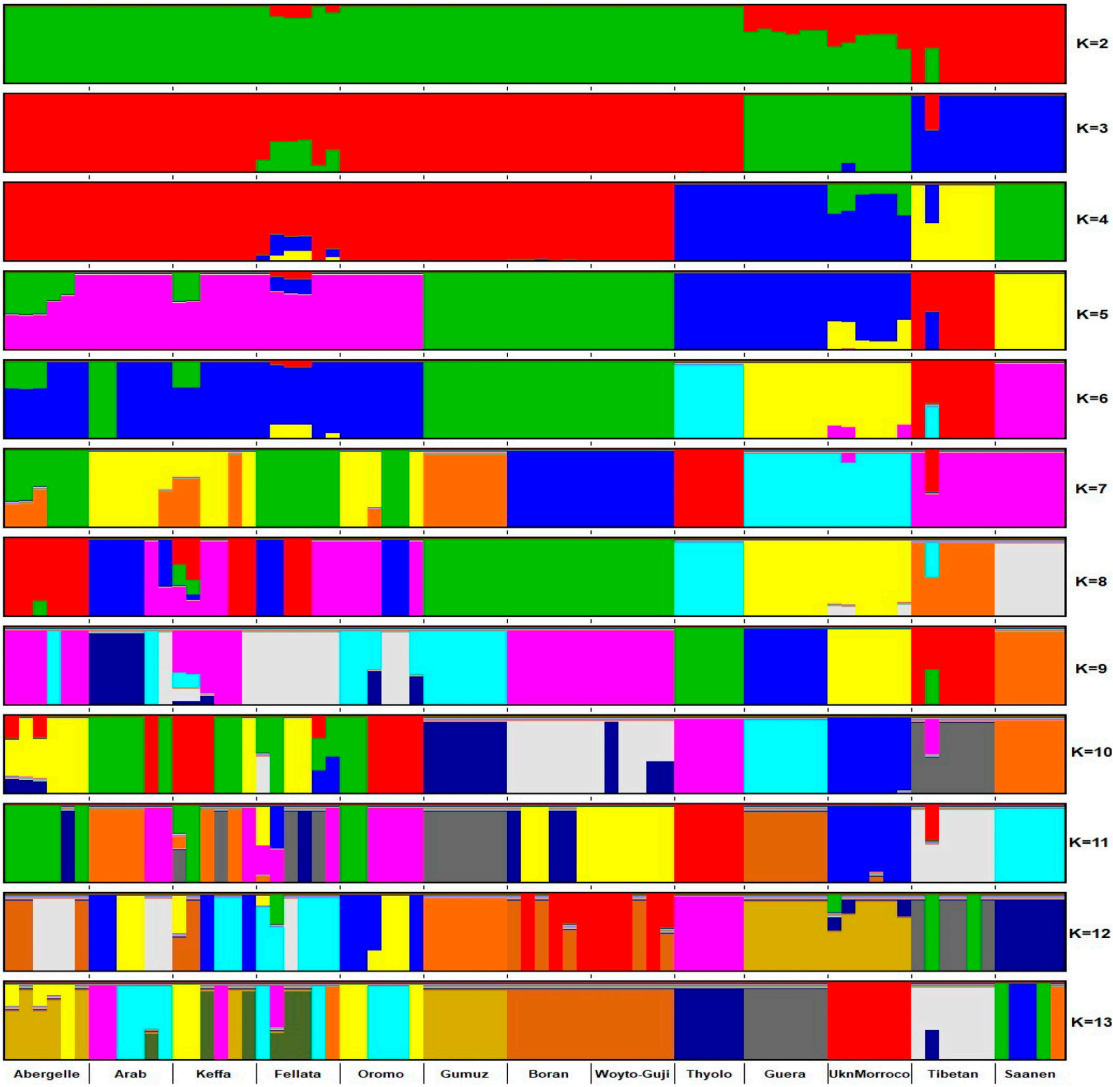
ADMIXTURE plot of the studied goat populations in a global context for 2 ≤ K ≤ 13 (EA-G1: East African Group 1; EA-G2: East African Group 2; NWSA: North, West and South African; AS: Asian; EU: European).

The graphical summary of the results of ADMIXTURE for 2 ≤ *K* ≤ 13 is presented in Figure 6. The cross-validation (CV) error was lowest at *K* = 6 (Supplementary Figure S2), suggesting the presence of six optimal genetic backgrounds in the 13 study goat populations. At this *K*-value, Saanen and Tibetan each show separate genetic backgrounds, suggesting they are genetically distinct. This separation is also revealed by the PCA (Figure 2) and Treemix (Figure 5). The Guera and Unknown breeds share the third genetic background, but the Unknown breed shows a small proportion of the Saanen background in its genome, which corresponds with the gene flow results from Saanen to Unknown in the TreeMix (Figure 5). Thyolo has the fourth genetic background, while Gumuz, Woyto-Guji, and Boran share the fifth genetic background. The sixth genetic background occurs in Abergelle, Keffa, Fellata, Arab, and Oromo. Some results are noteworthy. At *K* = 5, Thyolo, Guerra, and Unknown share a common genetic background, but Thyolo is separate with a different background at *K* = 6–13. The TreeMix also separates Thyolo and shows that it is closer to the populations from Ethiopia and Kenya (Figure 5). Similarly, the PCA completely separates Thyolo, which corresponds with the ADMIXTURE results for *K* = 6–13 (Figure 3). At *K* = 6, the background that defines Gumuz, Woyto-Guji, and Boran is observed in Abergelle, Arab, and Keffa, while Fellata shows the presence of the Tibetan background and the background defining Guera and Unknown in its genome. At *K* = 7, Gumuz diverges from Boran and Woyto-Guji, but its genetic background is now observed in Abergelle, Arab, Keffa, and Oromo. At this *K-*value, Fellata shares a background with Abergelle and Oromo goats but is separate from Keffa and Arab (Figure 6). This separation is also revealed by both PCA (Figure 4) and TreeMix (Figure 5). Abergelle, on the other hand, clusters with Arab, Keffa, and Oromo in both PCA and TreeMix, but ADMIXURE shows Abergelle with a different genetic background at *K* = 7–13, which it shares with Fellata. Arab, which clusters with Abergelle, Keffa, and Oromo in PCA (Figure 4) and TreeMix (Figure 5), shares some genetic background with these populations at K = 7–13 in ADMIXTURE. Gumuz clusters with Woyto-Guji and Boran in PCA (Figures 3, 4) but separates from Woyto-Guji and Boran in TreeMix (Figure 5). This separation is also revealed by ADMIXTURE at *K* = 5, 6, and 8 but not at *K* = 7 and *K* = 9–13.

At *K* = 6, six genetic clusters were observed. These are designated as Cluster 1, which comprised Arab, Fellata, Oromo, Abergelle, and Keffa; Cluster 2, which included Gumuz, Woyto-Guji, and Boran; Cluster 3 consisted of Thyolo; Cluster 4 embraced Unknown and Guera; and Tibetan and Saanen comprised their own individual clusters.

### Selection signatures—*F*_*ST*_ approach

**FIGURE 7:**
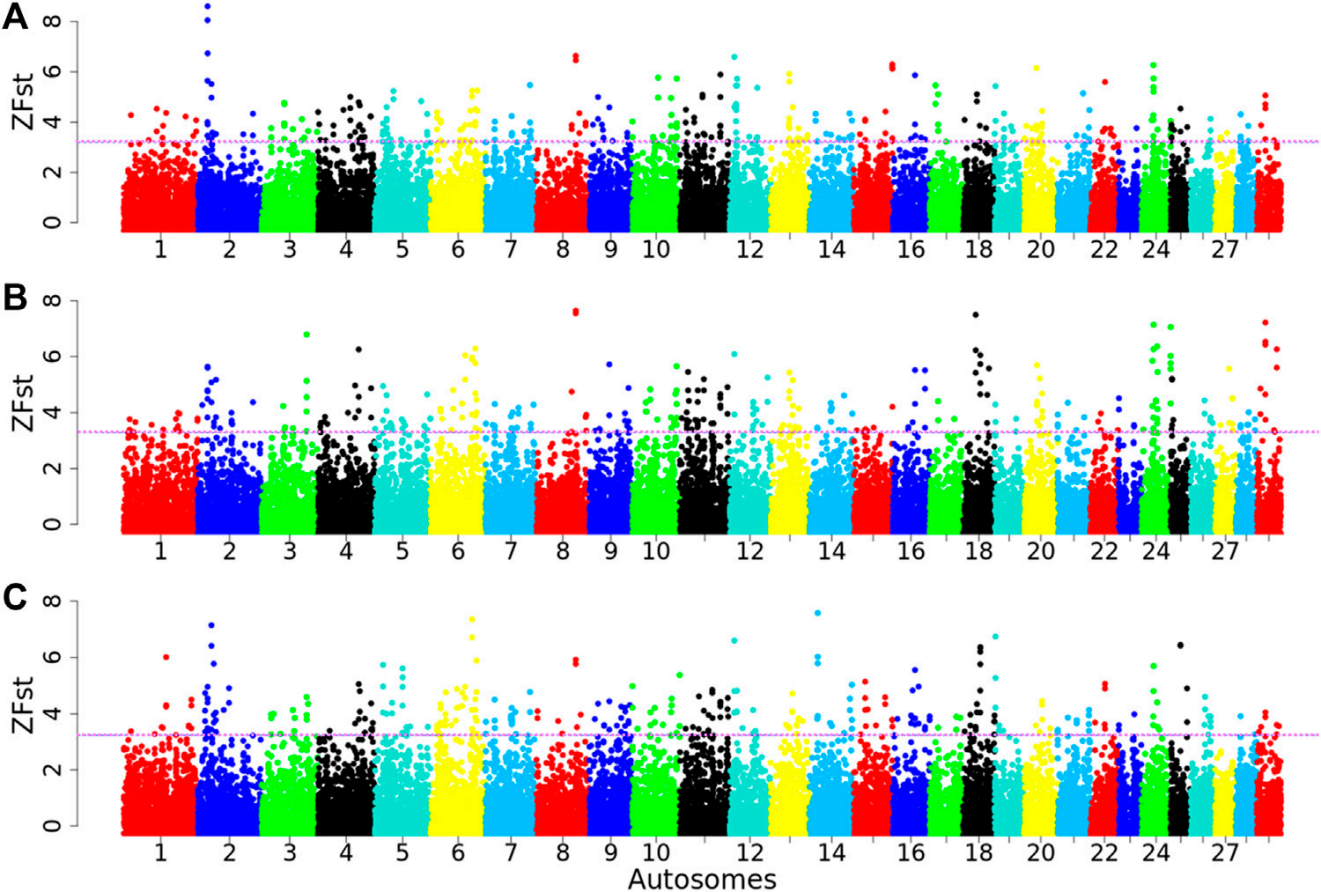
Manhattan plots for selection sweep analysis **(A)** between Arab vs. Tibetan, **(B)** between Fellata vs. Tibetan, and **(C)** between Oromo vs. Tibetan goat populations performed using the standardized population differentiation (*ZF* *ST*) approach. The horizontal line represents the arbitrary threshold for *ZFst*.

A total of 98,660 chromosome windows were assessed for each pairwise comparison (Arab vs. Tibetan, Fellata vs. Tibetan, and Oromo vs. Tibetan). The genome-wide distributions of the standardized *FST* values across the genomes for each goat population are shown in Supplementary Figure S3. The average *FST* values were 0.14, 0.11, and 0.13 in Arab, Fellata, and Oromo goats, respectively, suggesting moderate differentiation among the three populations, and this provides insights into their common genetic backgrounds and possible gene flow. Using the top 1% outlier windows as a cutoff threshold (as described earlier) in each population, ∼987 windows were assessed per population. Accordingly, genomic regions with high *ZF*_*ST*_ (*ZF*_*ST*_ ≥ 3.20 (range = 3.20–8.22), 3.29 (range = 3.29–7.96), and 3.24 (range = 3.24–7.34); corresponding *F*_*ST*_ ≥ 0.40, 0.35, 0.40) in the Arab vs. Tibetan, Fellata vs. Tibetan and Oromo vs. Tibetan, respectively, were defined as selection signatures (Figure 7). Based on these criteria, 987 genomic regions were detected for each of the Arab, Fellata, and Oromo populations (Supplementary Tables S6–S8). Among the three populations, there was variation in the distribution of the regions across the genome. In the Arab and Oromo populations, chromosome 2 showed a strong selection signal, whereas in the Fellata population, chromosomes 8 and 18 showed stronger sweeps than the other chromosomes (Figure 7).

### Selection signatures—*Hp* approach

**FIGURE 8:**
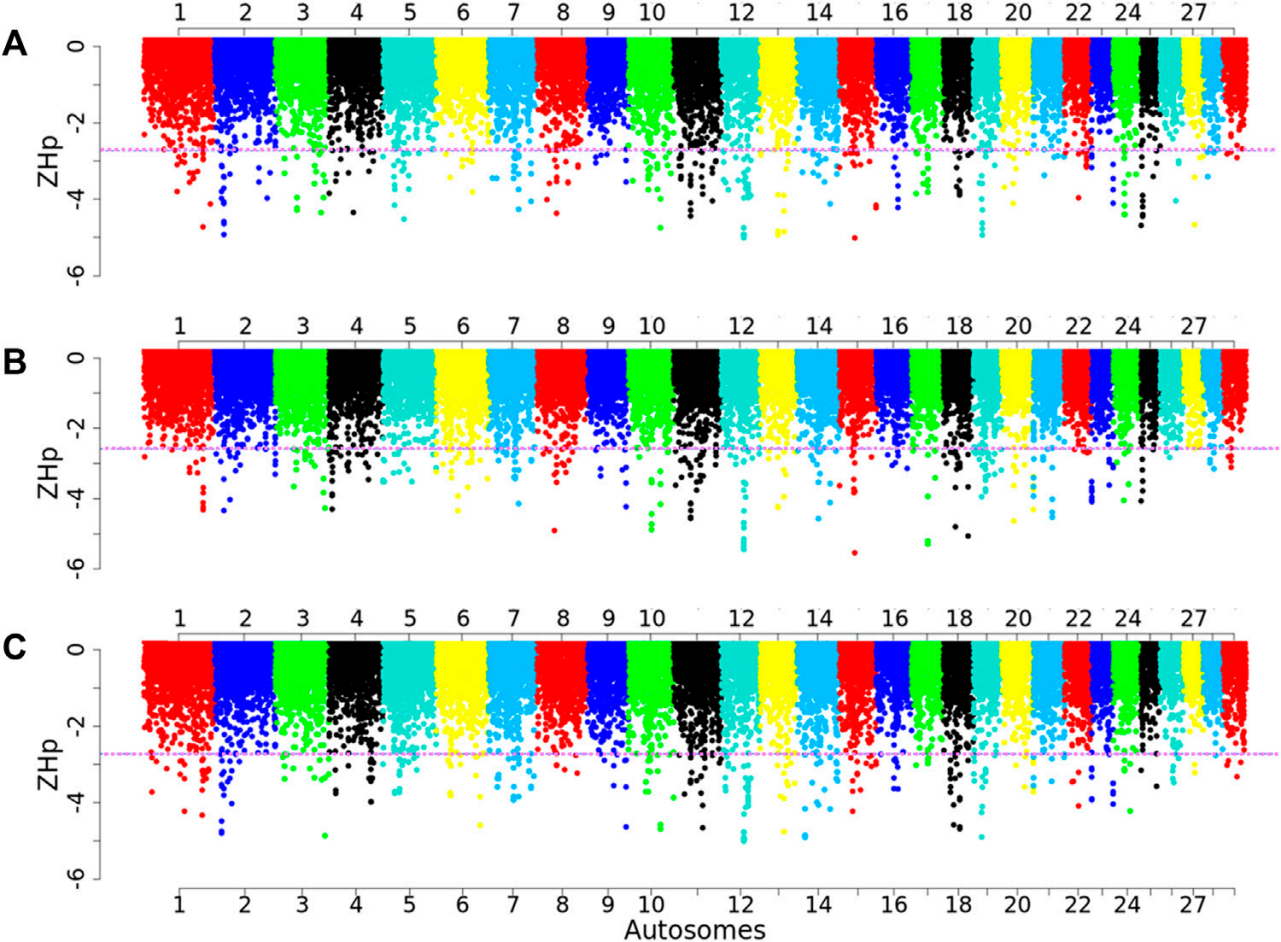
Manhattan plots performed using the standardized pool heterozygosity (*ZHp*) approach for each 100-kb sliding window with a 25-kb step size across all autosomes in the **(A)** Arab, **(B)** Fellata, and **(C)** Oromo goat populations. The horizontal line represents the arbitrary threshold for *ZHp*.

We also calculated the Z-transformed pooled heterozygosity (*ZHp*) in 100-kb sliding windows and a 25-kb step size to detect selection sweeps in the three Ethiopian indigenous goat populations. Accordingly, 98,573 windows were detected per population. The overall average *Hp* values across all the windows were 0.19, 0.19, and 0.18 in the Arab, Fellata, and Oromo goats, respectively (Supplementary Figure S4). Similarly, only outliers falling within the bottom 1% (∼986 windows) with low *ZHp* values (ZHp ≤ −2.73, −2.59, and −2.72; corresponding *Hp* ≤ 0.08, 0.09, and 0.08 in the Arab, Fellata, and Oromo goats, respectively, were considered signatures of selection. Accordingly, ∼983 genomic regions were detected in each population (Supplementary Tables S9–S11). Figure 8 displays the Manhattan plot of the *ZHp* values for several comparisons in each population. In both the Arab and the Fellata goats, chromosome 15 was shown to have the highest signs for selection signatures, while chromosome 14 was detected in the Oromo population.

### Overlapping selection signature regions, genes identified, and enrichment analysis

Based on the overlap of the top 1% *ZF*_*ST*_ and the bottom 1% *ZHp*, 250, 174, and 285 putative selection signature regions were detected for Arab, Fellata, and Oromo goats, respectively (Supplementary Tables S12–S14). Supplementary Tables S15–S17 show a set of functional genes that are novel and reported after annotation of those overlapping genomic regions for Arab, Fellata, and Oromo goat populations, respectively. A total of 206 genes within 65 putative selection signature regions were detected to be associated with Arab goats (Supplementary Table S15). Similarly, 107 genes within 42 candidate genomic regions were identified in Fellata goats (Supplementary Table S16), and 195 genes within 63 genomic regions were identified in Oromo goats (Supplementary Table S17). In total, 508 genes were detected within 170 regions for the three goat populations (Supplementary Tables S15–S17). Among the 170 candidate regions, nine regions were the strongest, including CHI5, CHI7, and CHI19 in Arab goats (Supplementary Table S15); CHI5, CHI7, and CHI18 in Fellata goats (Supplementary Table S16); and CHI5, CHI15, and CHI19 in Oromo goats (Supplementary Table S17). These nine regions spanned a total of 163 candidate genes (Supplementary Tables S15–S17). Enrichment analysis was performed with the 163 genes using DAVID on the goat gene set (*C. hircus*) with default settings. Based on the annotation, of the 163 genes, 125 were significantly (*p* ≤ 0.05, Bonferroni correction) enriched, and we found nine biological process (BP) terms, five cellular component (CC) terms, six molecular function (MF) terms, and one KEGG pathway (Supplementary Tables S18–S21).

## Discussion

In the present study, we investigated the genome architecture of three northwestern populations of Ethiopian indigenous goats and benchmarked them against other goat populations from eastern, western, northern, and southern Africa, as well as two exotic breeds, Saanen and Tibetan, from Europe and Asia, respectively. The overall average Ho and He exceeded 0.322, and the within-population genetic variation (D) was above 0.278, suggesting the study populations are highly genetically diverse. The values for Ho and He observed in this study are within the range of global goat diversity (Colli et al., 2018) and close to those reported in Sudanese (Rahmatalla et al., 2017) and Pakistani (Kumar et al., 2018) goats and in the Egyptian Barki goat breed (Kim et al., 2016). The higher variability observed within the Ethiopian goat populations could be attributed to uncontrolled mating that occurs as a result of the common practice of flocks utilizing communal grazing and watering points. The absence of artificial selection, high levels of admixture in these populations, and the introduction and crossbreeding of exotic goats into Ethiopia can also be possible explanations for the increased heterozygosity. Artificial selection within a population may benefit from high variation within populations, especially in areas where community-based goat breeding programs (CBBP) are practiced. CBBP, in which artificial selection occurs within a population, provides a good framework for the implementation of genomic selection in smallholder production systems (Mrode et al., 2018; Rekik et al., 2021).

Runs of homozygosity (ROH) are two contiguous identical by descent (IBD) genomic segments (Gibson et al., 2006) that arise from an increased level of relatedness between individuals within a population or through positive selection (Kijas, 2013). Estimates of ROHs can be used to assist with the interpretation of genomic inbreeding and give insights into population history (Purfield et al., 2012; Zavarez et al., 2015). The latter is particularly important for African indigenous livestock, which are characterized by a lack of written pedigree data (Kosgey et al., 2006). According to Purfield et al. (2012), short ROHs are most likely correlated to ancient inbreeding or potential ancient bottlenecks, whereas long ROHs are more likely associated with relatively recent inbreeding. In the present study, the Ethiopian indigenous goat populations showed their majority of ROHs in the short (100–150 Kb) length category, which is in agreement with the results obtained for other goats (Kim et al., 2016; Brito et al., 2017; Onzima et al., 2018; Islam et al., 2019). The accumulation of ROHs in the short-length category indicates that the study goats could have been initially established by small founding populations but were not particularly affected by recent inbreeding.

A combination of PCA, ADMIXTURE, and TreeMix tools provided an insight into the genetic structure of the three indigenous goat populations from northwestern Ethiopia and referenced other Ethiopian indigenous goat populations as well as goat populations from Africa and one each from Europe and Asia. The ADMIXTURE tool revealed two distinct genetic clusters. At K = 5, Gumuz and Woyto-Guji were grouped together, while Abergelle, Arab, Fellata, Keffa, and Oromo were in a different cluster and showed some degree of admixture. This result was supported by the TreeMix result. Our finding on these two genetic groups mirrors the previous findings from mitochondrial DNA analyses of 13 Ethiopian goat populations, which identified two haplogroups (A and G) (Tarekegn et al., 2018), suggesting the presence of two deep ancient ancestries in Ethiopia. However, based on the current dataset, it is difficult to infer whether the two groups introduced/arrived in Ethiopia together or independently. The Gumuz and Woyto-Guji populations are geographically isolated (e.g., Gumuz is located in northwestern while Woyto-Guji is located in southern Ethiopia), but they clustered together and were separate from other Ethiopian goat populations. This could be due to their unique genetic compositions and similarity of production environments. We found that the heterozygosity values for Gumuz and Woyto-Guji were very similar (Ho = 0.334 ± 0.025 and 0.335 ± 0.009 for Gumuz and Woyto-Guji, respectively). This was further confirmed by comparable inbreeding coefficient and number of ROHs. The goats also inhabit similar agro-ecology (semi-arid), and both of them are kept by agro-pastoralists. The clustering of Arab, Fellata, and Oromo goats in one group is attributed to geographical proximity. Home tracts of the three goat populations tend to overlap, which may facilitate ease of flock exchange between farmers and favor gene flow among the populations. In general, the study goat populations are characterized by a high level of admixture and a lack of phylogeographic structure, and this agrees with Tarekegn et al. (2018), Luikart et al. (2001), and Naderi et al. (2007; 2008), who reported similar findings in different goat breeds.

We did find a total of 170 overlapping genomic regions in the three populations, spanning 508 candidate genes, by combining the two approaches. Comparable results have been reported for livestock species from similar environments (Kim et al., 2016; Mwacharo et al., 2017; Ayalew et al., 2023). Nine of 170 regions were identified as the strongest signals and covered 163 functional genes. We speculate that these 163 genes represent past and/or on-going selection in the studied goat populations. Many of the 163 candidate genes were associated with diverse physiological, molecular, and cellular processes and pathways (Supplementary Tables S18–S21). This shows that adaptation to semi-arid tropical environmental stressors such as heat, solar radiation, physical exhaustion, feed and water scarcity, parasites, and others is complex and may involve many genomic regions and genes with pleiotropic activities. It also demonstrates the intricacy of adaptation, involving numerous biological processes and quantitative trait loci, each of which has a small but cumulative effect on the overall phenotypic expression. Among the significant GO terms, the top three that are related to tropical environment adaptations include anterior/posterior pattern specification (GO:0009952), positive regulation of natural killer cell proliferation (GO:0032819), and positive regulation of natural killer (NK) T cell proliferation (GO:0051142) (Supplementary Table S18). Based on their biological functions and information from published studies, several genes that have been reported before and are possibly responsible for the important traits in goats and other domestic livestock were presented.

### Thermo-tolerance genes

Numerous inherent genetic endowments in the Ethiopian indigenous goat populations could be harnessed for better adaptation in semi-arid tropical environments. We identified some of these genetic biomarkers, notably the *HOXC-*cluster (homeobox genes) (*HOXC12, HOXC13, HOXC4, HOXC6*, and *HOXC9*) and *MAPK8IP2* (Supplementary Table S18), that are associated with heat stress. The *HOXC-*cluster was present in Arab and Oromo goat populations and was also pinpointed by GO analysis (GO:0009952), revealing the term anterior/posterior pattern specification. Onzima et al. (2018) reported *HOXC12* and *HOXC13* genes for their involvement in anterior/posterior pattern specification in the Sebei goat breed of Uganda. The genes can also regulate essential traits like keratin and hair follicle differentiation in goats (Wu et al., 2009), sheep (Sander and Powell, 2004), and cattle (Taye et al., 2017; Ayalew et al., 2023). More specifically, the *HOXC13* gene has been reported to influence skin thickness and number of hair follicles in animals. Skin, being the intermediate between the animal body and the surrounding environment, influences thermoregulation positively (Alfonzo et al., 2016) and plays a major role in aiding the adaptation of animals to heat stress. Cattle with relatively thicker skin, such as the thermo-tolerant *Bos indicus*, exhibit better thermoregulation than cattle with thinner skin, like the heat-sensitive *Bos taurus* (Alfonzo et al., 2016). The present study identifies *HOXC13* as one of the biomarkers for thermo-tolerance, which could further aid in long-term breeding goals towards developing agro-ecological zone-specific goat breeds in the study area. The other gene, *MAPK8IP2,* is identified in the candidate genomic region of the Fellata goat population, and it has been found to be involved in different aspects of thermo-tolerance. This gene has been shown to interact with *MAPK8IP1* (Yasuda et al., 2000). The latter is involved in regulating the reaction of cells to heat stress, which raises the transcription activity of multiple heat stress-responsive genes that regulate various processes, such as cell survival, proliferation, and apoptosis in Holstein cows involved in milk production under heat stress (Sigdel et al., 2019). When cells are subjected to heat stress, they release reactive oxygen species (ROS), which result in cellular necrosis and, ultimately, cell death. Interestingly, gene *MAPK8IP1* is involved in suppressing heat stress-induced ROS production and cellular apoptosis (Li et al., 2018).

### Immune response genes

Indigenous goats are known to be well-adapted to various environmental stressors, including disease. A member of interleukins (IL18) and TYK2 were present in one of the candidate regions identified in Arab and Oromo goat populations. Interleukins are expressed by leukocytes (Maggio et al., 2006) and are likely involved in the activation of the immune response of goats (Amiri et al., 2023) and chickens (Susta et al., 2015; Chen et al., 2016; Truong et al., 2016). In a study conducted on Malabari goats during heat stress, IL18 was identified as a reliable immunological marker that aids in assessing heat stress-mediated immune response alterations (Rashamol et al., 2019). Similarly, Madhusoodan et al. (2019) reported a significant reduction in hepatitic IL18 gene expression during heat stress in Salem black goats (Madhusoodan et al., 2019). This gene is considered an important inflammatory marker for quantifying the impact of heat stress on the immune system in goats (Bagath et al., 2019). Similarly, *TYK2* plays a vital role in the innate and acquired responses of humans (Lu et al., 2015), including inflammatory conditions resulting from viral infections and autoimmune diseases. Some other important genes identified in the present study, known to be associated with immune response, are members of *ICAM,* such as *ICAM3* in Arab and Oromo goat populations and subfamily members of *ADGRG,* including *ADGRG1* and *ADGRG3* in Fellata goats. *ICAM3*, the gene constitutively expressed on the surface of leukocytes (Xiao et al., 2013), is important in generating an immune cell response (Montoya et al., 2002) through its facilitation of interactions between T cells and dendritic cells (*Svajger et al., 2010*). The other gene, *ADGRG1*, plays a role in immune regulation (Lala and Hall, 2022) and is expressed in cytotoxic lymphocytes, including natural killer (NK) cells (Della Chiesa et al., 2010). *ADGRG3* has been shown to exert robust effects on B cell development (Wang et al., 2013). Genetic deletion of *ADGRG3* reduces macrophage migration into white adipose tissue (Shi et al., 2016). The fact that *ADGRG3* is robustly expressed in multiple immune cells is fascinating with regard to the recent revelation that *ADGRG3* can be activated by glucocorticoids (Ping et al., 2021), as glucocorticoids are known to exert powerful effects on the physiology of many different cell types in the immune system.

### Genes associated with production and reproduction

We detected genes like *RARG* and *DNMT1* in Arab and Oromo goats, which are associated with production and reproduction. *RARG* plays an important role in milk production, body size, and kidding in Liaoning cashmere goats (Chen et al., 2022). The expression of this gene has been suggested to be required for the development of limb buds and skeletal growth in dairy cows (Bionaz et al., 2015). Furthermore, *RARG* was identified as the most important master regulator of quantitative trait loci (QTL) for milk production in the F_2_ population of German Holstein × Charolais crossed cows (Brand et al., 2016). This gene was also associated with litter size in pigs (Rothschild et al., 1997) and the development of bovine embryos (Mohan et al., 2001). In other species, such as mice, *RARG* was reported to be of functional significance involved in spermatogenesis (Aurore et al., 2012), formation of normal alveoli and alveoli elastic fibers in the lung (McGowan et al., 2000), hematopoietic development (Purton, 2007), and correct formation of the axial skeleton, including anteriorization of the cervical and thoracic vertebrae (Wendling et al., 2001). In contrast, its absence in mice resulted in growth deficiency (Lohnes et al., 1993), bone mass reduction (Green et al., 2015), reduced chondrocyte proliferation, decreased expression and deposition of proteo1glycans (Williams et al., 2009), and male sterility (Lohnes et al., 1993). The other gene, *DNMT1,* consistently appears to be involved in reproduction (Kay et al., 2018; Schulz et al., 2018; Bewick et al., 2019; Amukamara et al., 2020; Washington et al., 2021) and egg production and egg viability in *B. tabaci*. *DNMT1* knockdown affected testis size and structure (Washington et al., 2021).

## Conclusion

We used whole-genome sequence data to investigate the level of genetic diversity, population structure, and signatures of selection in three Ethiopian indigenous goat populations. We observed high within-breed genetic diversity, low genetic differentiation, a high level of admixture, and lack of phylogeographic structure in the studied goat populations. The genomic data also identified several potential candidate genes possibly under selection, including adaptation to a hot environment (homeobox genes and *MAPK8IP2*), immune response (*IL18, TYK2, ICAM3,* and *ADGRG* subfamilies), and production and reproduction (*RARG* and *DNMT1*). Our study is important in the design of goat genetic improvement programs in view of the current and future predicted effects of climate change. It also provides a foundation for future studies to investigate the genome architectures of different ruminant species coexisting in a similar environment.

## Data Availability

The datasets presented in this study can be found in online repositories. The names of the repository/repositories and accession number(s) can be found below: NCBI SRA database (accession number PRJNA1063878).
