# Supplementary figures and images for: Whole-genome resequencing reveals genomic variation and dynamics in Ethiopian indigenous goats
Source: Front Genet. 2024 May 24;15:1353026. doi: 10.3389/fgene.2024.1353026 (PMC11156998; doi:10.3389/fgene.2024.1353026)

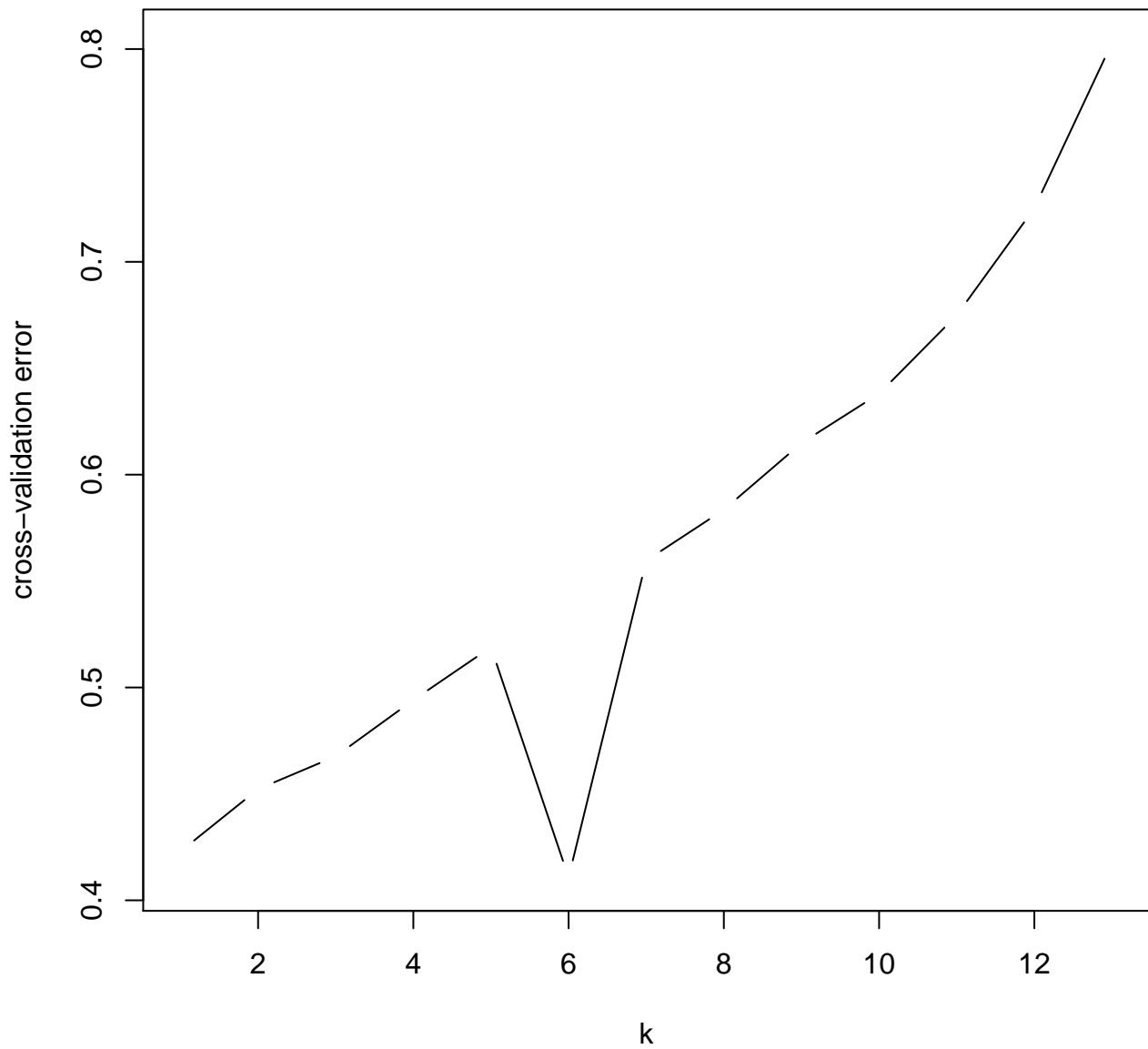

Supplement: Supplementary file 1 [file DataSheet2.PDF]

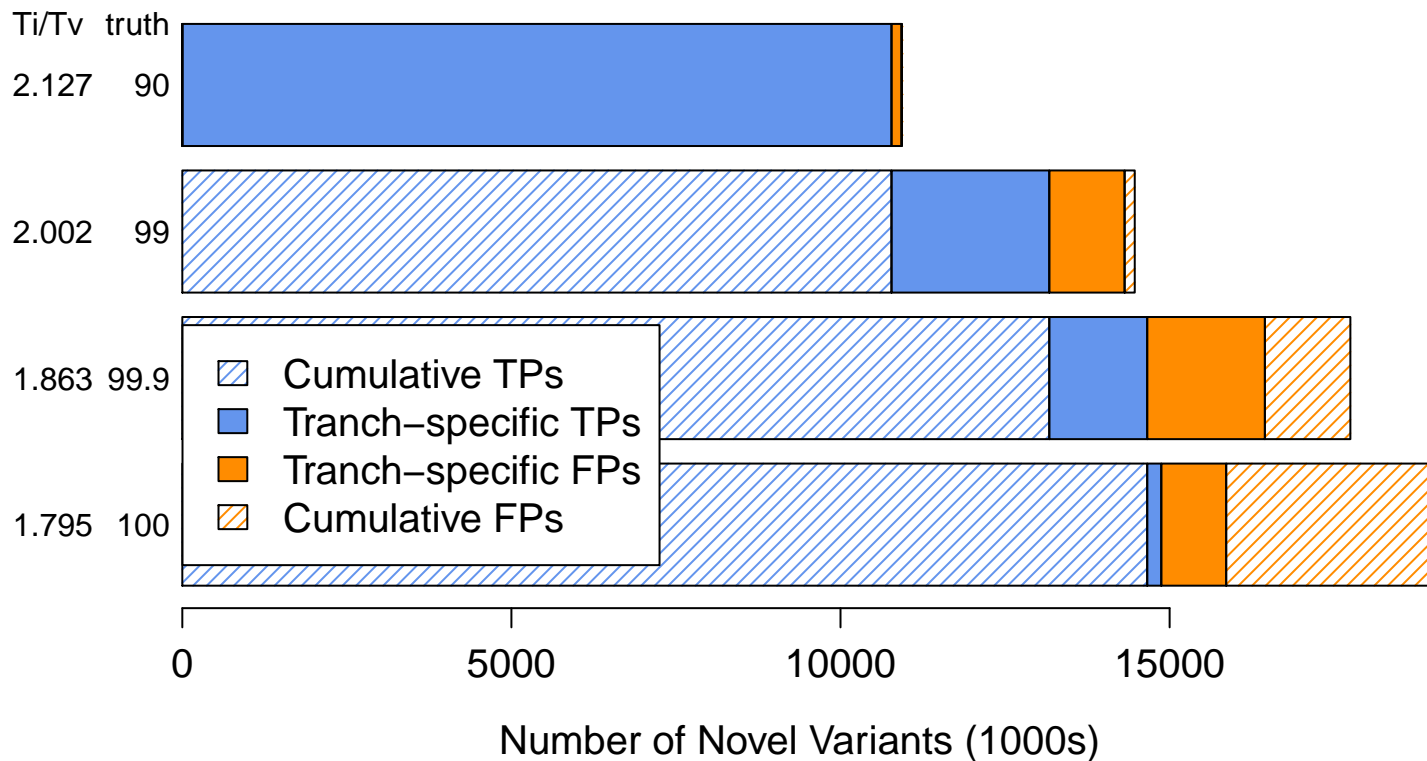

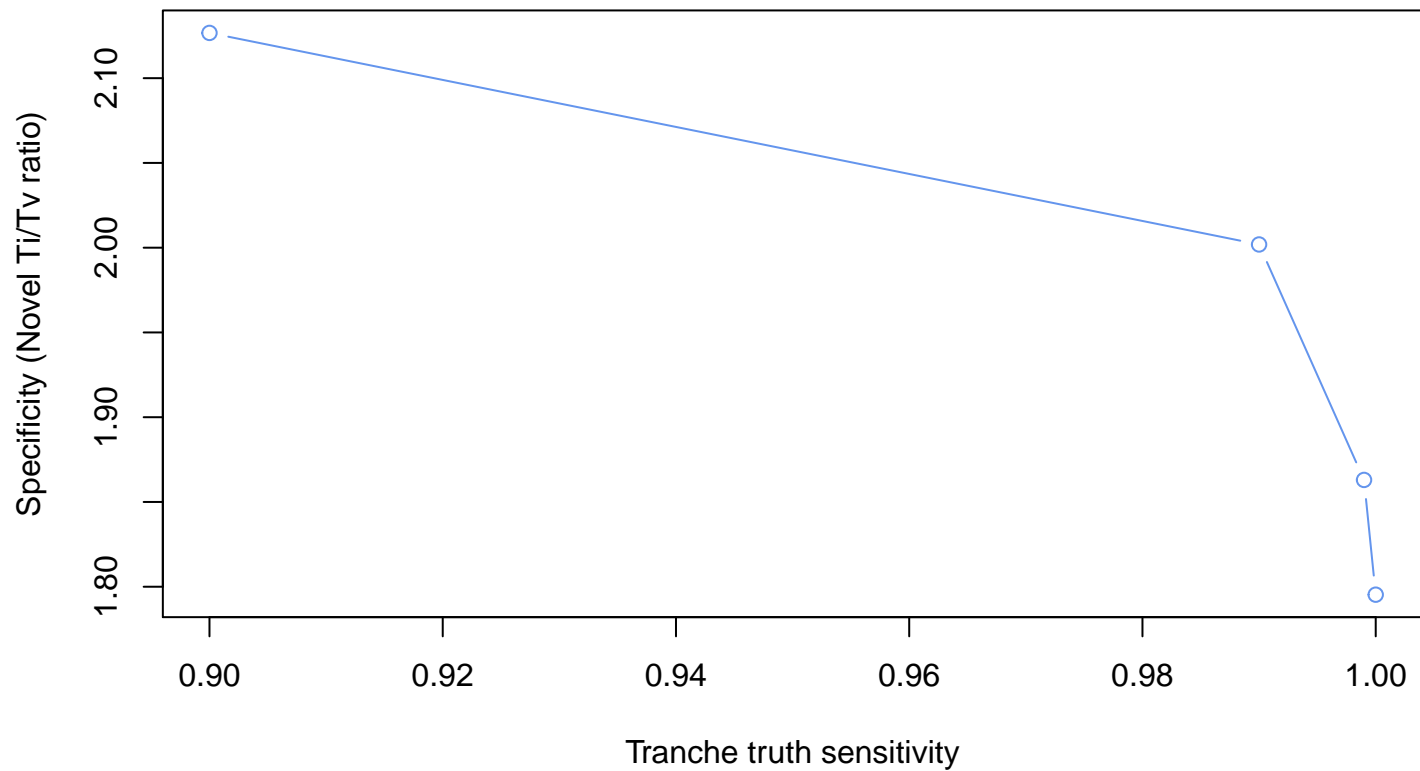

Supplement: Supplementary file 6 [file DataSheet1.PDF]
