# Supplementary material for: Whole-genome resequencing reveals genomic variation and dynamics in Ethiopian indigenous goats
Source: Front Genet. 2024 May 24;15:1353026. doi: 10.3389/fgene.2024.1353026 (PMC11156998; doi:10.3389/fgene.2024.1353026)
Supplement: Supplementary file 2 [file Table1.DOCX]

**Supplementary Table S1**: Description of the environmental characteristics at the level of the distribution area of the study goat populations

| **Breed name** | **Country** | **Area of the breed in the country** | **Climatic characteristics of the distribution area** | **Sample location (latitude/ longitude)** |
| --- | --- | --- | --- | --- |
| Arab | Ethiopia | Semi-arid: Oumer et al., 2019 | Mean altitude: 1,373 m  Annual mean temperature: 20–30 °C | 6.44/37.92 |
| Fellata | Ethiopia | Hot arid: Getinet et al., 2005 | Mean altitude: 725 m  Annual mean temperature: 19–38.7 °C | 10.34/34.22 |
| Oromo | Ethiopia | Sub-humid: Oumer et al., 2019 | Mean altitude: 1,668 m  Annual mean temperature: 28 °C | 9.45/34.44 |
| Abergelle | Ethiopia | Warm sub-moist lowland: Birhanie et al., 2018 | Mean altitude: <1,500 m  Annual mean temperature: 21–41 °C | 13.09/38.95* |
| Keffa | Ethiopia | Humid and forest area: Getinet, 2016 | Mean altitude: 1,814 m  Annual mean temperature: 10–27.5 °C | 5.87/36.47* |
| Gumuz | Ethiopia | Hot arid: Getinet et al., 2005 | Mean altitude: 630 m  Annual mean temperature: 23.5–30 °C | 9.15/40.49* |
| Woyto-Guji | Ethiopia | Semi-arid to sub-humid: Zergaw et al., 2016 | Mean altitude: 600–1,200 m  Annual mean temperature: 12–33 °C | 6.86/37.81* |
| Galla: GAL | Kenya | Northern arid and semi-arid: Porter et al., 2016 | Mean altitude: 590.6 m  Annual mean temperature: 26.7 °C | -0.02/37.91* |
| Unknown | Morocco | http://boujenane.com/phocadownload/Small%20ruminant%20breed%20in%20Morocco.pdf | Mean altitude: 1,500 m  Annual mean temperature: 14–22 °C | 31.79/-7.09* |
| Thyolo | Malawi | Semi-arid: Banda et al., 2009 | Mean altitude: 820 m  Annual mean temperature: 29 °C | -13.25/34.30* |
| Guera | Mali | Semi-arid: Traoré et al., 2021 | Mean altitude: 850 m  Annual mean temperature: 16–22 °C | 16.56/-1.62* |
| Saanen | France | Cool: https://en.wikipedia.org/wiki/French_Alps | Mean altitude: 1,125–4,810 m  Annual mean temperature: -8°C | 45.92/7.04* |
| Tibetan | China | Cool: https://www.britannica.com/place/Tibet/Climate | Mean altitude: 4,800–6,100 m  Annual mean temperature: -19–30°C | 30.15/88.79* |

* According to Bertolini et al. (2018): mean altitude in meters, annual mean temperature in degree Celsius and annual precipitation in millimeters.
