# Supplementary material for: Whole-genome resequencing reveals genomic variation and dynamics in Ethiopian indigenous goats
Source: Front Genet. 2024 May 24;15:1353026. doi: 10.3389/fgene.2024.1353026 (PMC11156998; doi:10.3389/fgene.2024.1353026)
Supplement: Supplementary file 4 [file Table7.DOCX]

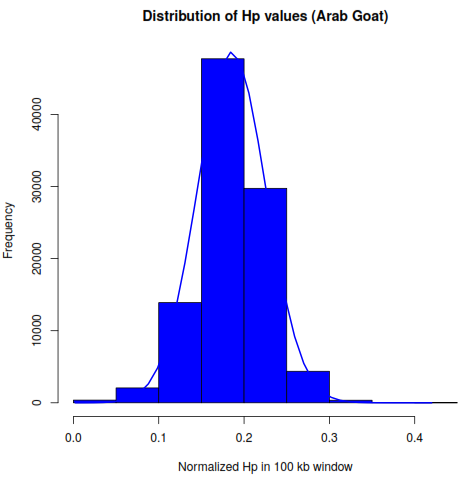


**
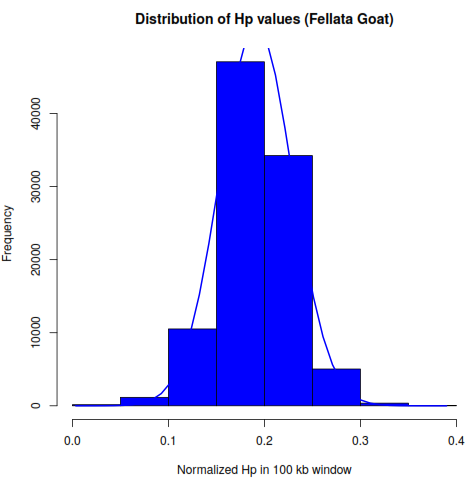
**

**
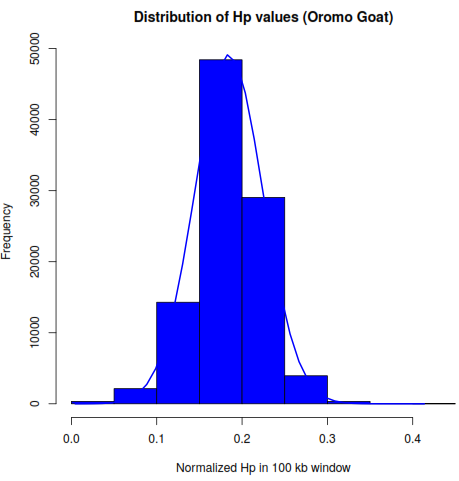
**

**Supplementary Figure F4:** Genome-wide distributions of *Hp* in the **(A)** Arab; **(B)** Fellata and **(C)** Oromo goat populationss.

**A**

**B**

**C**
