# Supplementary material for: Whole-genome resequencing reveals genomic variation and dynamics in Ethiopian indigenous goats
Source: Front Genet. 2024 May 24;15:1353026. doi: 10.3389/fgene.2024.1353026 (PMC11156998; doi:10.3389/fgene.2024.1353026)
Supplement: Supplementary file 5 [file Table2.DOCX]

**Supplementary Table S2:** SNP statistics for each goat population

| **Population** | **Total number of SNPs** | **Average per sample** | **Reported SNPs (rs)** | **Novel SNPs** | **het/hom** | **dbSNP (%)** | |
| --- | --- | --- | --- | --- | --- | --- | --- |
|  |  |  |  |  |  | **rs** | **novel** |
| ARB | 15,878,802 | 8,078,778 | 10,891,308 | 4,987,494 | 1.27 | 68.59 | 31.41 |
| FEL | 16,903,989 | 7,983,054 | 11,648,111 | 5,255,878 | 1.32 | 68.91 | 31.09 |
| ORO | 15,889,419 | 7,989,569 | 10,900,991 | 4,988,428 | 1.27 | 68.61 | 31.39 |
| ABR | 16,408,691 | 7,701,195 | 10,851,974 | 5,556,717 | 1.34 | 66.14 | 33.86 |
| KEF | 14,936,598 | 6,591,579 | 10,173,862 | 5,826,286 | 1.03 | 68.11 | 31.89 |
| GUM | 16,682,139 | 8,751,774 | 11,294,508 | 5,387,631 | 1.44 | 67.70 | 32.30 |
| WGU | 17,262,645 | 8,866,137 | 11,677,476 | 5,585,169 | 1.55 | 67.65 | 32.35 |
| GAL | 17,400,421 | 8,781,270 | 11,783,862 | 5,616,559 | 1.57 | 67.72 | 32.28 |
| UNK | 18,266,925 | 8,589,128 | 12,986,129 | 5,280,796 | 1.62 | 71.09 | 28.91 |
| THY | 14,750,501 | 8,281,573 | 9,945,667 | 4,804,834 | 1.14 | 67.43 | 32.57 |
| GUE | 15,853,996 | 8,075,373 | 10,885,132 | 4,968,864 | 1.39 | 68.66 | 31.34 |
| SAN | 14,389,837 | 7,225,373 | 9,841,654 | 4,548,183 | 1.15 | 68.39 | 31.61 |
| TIB | 17,459,764 | 8,346,515 | 11,412,217 | 6,047,547 | 1.31 | 65.36 | 34.64 |
