# Supplementary material for: Whole-genome resequencing reveals genomic variation and dynamics in Ethiopian indigenous goats
Source: Front Genet. 2024 May 24;15:1353026. doi: 10.3389/fgene.2024.1353026 (PMC11156998; doi:10.3389/fgene.2024.1353026)
Supplement: Supplementary file 7 [file Table5.DOCX]

**Supplementary Table S5:** Results of f4 statistics for the study populations as generated with TreeMix

| **S. No** | **Breeds (A,B; C,D)** | **f4-statistics** | **Standard Error** | **Z** |
| --- | --- | --- | --- | --- |
| 1 | Galla,Abergelle;Saanen,Gumuz | -0.0007847 | 4.44E-05 | -17.687 |
| 2 | Galla,Gumuz;Abergelle,Saanen | -0.000349352 | 4.40E-05 | -7.93399 |
| 3 | Galla,Saanen;Abergelle,Gumuz | -0.00113405 | 4.43E-05 | -25.5839 |
| 4 | Galla,Abergelle;Saanen,Keffa | 0.000871779 | 4.14E-05 | 21.0446 |
| 5 | Galla,Keffa;Abergelle,Saanen | 0.000139987 | 4.34E-05 | 3.22692 |
| 6 | Galla,Saanen;Abergelle,Keffa | 0.00101177 | 3.93E-05 | 25.7639 |
| 7 | Galla,Abergelle;Saanen,Thyolo | -0.00130601 | 4.87E-05 | -26.8401 |
| 8 | Galla,Saanen;Abergelle,Thyolo | -0.000183427 | 5.52E-05 | -3.32195 |
| 9 | Galla,Thyolo;Abergelle,Saanen | 0.00112258 | 5.39E-05 | 20.8445 |
| 10 | Galla,Abergelle;Saanen,Fellata | -0.000351422 | 3.90E-05 | -9.01638 |
| 11 | Galla,Fellata;Abergelle,Saanen | 0.00198217 | 4.21E-05 | 47.1173 |
| 12 | Galla,Saanen;Abergelle,Fellata | 0.00163075 | 4.32E-05 | 37.7196 |
| 13 | Galla,Abergelle;Saanen,Arab | 0.000166463 | 4.25E-05 | 3.91882 |
| 14 | Galla,Arab;Abergelle,Saanen | 0.000171487 | 4.45E-05 | 3.85494 |
| 15 | Galla,Saanen;Abergelle,Arab | 0.000337951 | 4.46E-05 | 7.57294 |
| 16 | Galla,Abergelle;Saanen,Guera | -0.000509008 | 4.08E-05 | -12.4822 |
| 17 | Galla,Guera;Abergelle,Saanen | 0.00457978 | 5.25E-05 | 87.181 |
| 18 | Galla,Saanen;Abergelle,Guera | 0.00407077 | 5.45E-05 | 74.6337 |
| 19 | Galla,Abergelle;Saanen,UknMorocco | -0.000331598 | 3.52E-05 | -9.43018 |
| 20 | Galla,Saanen;Abergelle,UknMorocco | 0.00717045 | 5.18E-05 | 138.432 |
| 21 | Galla,UknMorocco;Abergelle,Saanen | 0.00750205 | 5.13E-05 | 146.232 |
| 22 | Galla,Abergelle;Saanen,Woyto-Guji | -0.00181323 | 4.27E-05 | -42.4521 |
| 23 | Galla,Saanen;Abergelle,Woyto-Guji | -0.00209513 | 4.25E-05 | -49.315 |
| 24 | Galla,Woyto-Guji;Abergelle,Saanen | -0.000281907 | 3.71E-05 | -7.60638 |
| 25 | Galla,Abergelle;Saanen,Oromo | 0.000398348 | 4.20E-05 | 9.48859 |
| 26 | Galla,Oromo;Abergelle,Saanen | -0.00010104 | 4.33E-05 | -2.33407 |
| 27 | Galla,Saanen;Abergelle,Oromo | 0.000297309 | 4.10E-05 | 7.25567 |
| 28 | Galla,Abergelle;Saanen,Tibetan | -4.54E-05 | 3.78E-05 | -1.201 |
| 29 | Galla,Saanen;Abergelle,Tibetan | 0.0085436 | 6.06E-05 | 141.097 |
| 30 | Galla,Tibetan;Abergelle,Saanen | 0.00858899 | 6.08E-05 | 141.322 |
| 31 | Galla,Abergelle;Gumuz,Keffa | 0.00165648 | 3.17E-05 | 52.234 |
| 32 | Galla,Gumuz;Abergelle,Keffa | 0.000361452 | 2.97E-05 | 12.1811 |
| 33 | Galla,Keffa;Abergelle,Gumuz | -0.00129503 | 3.34E-05 | -38.7495 |
| 34 | Galla,Abergelle;Gumuz,Thyolo | -0.000521311 | 3.91E-05 | -13.3381 |
| 35 | Galla,Gumuz;Abergelle,Thyolo | 3.13E-05 | 4.09E-05 | 0.766482 |
| 36 | Galla,Thyolo;Abergelle,Gumuz | 0.000552647 | 4.06E-05 | 13.6005 |
| 37 | Galla,Abergelle;Gumuz,Fellata | 0.000433278 | 3.37E-05 | 12.8637 |
| 38 | Galla,Fellata;Abergelle,Gumuz | -0.00048681 | 3.20E-05 | -15.208 |
| 39 | Galla,Gumuz;Abergelle,Fellata | -5.35E-05 | 3.26E-05 | -1.63961 |
| 40 | Galla,Abergelle;Gumuz,Arab | 0.000951163 | 3.29E-05 | 28.8679 |
| 41 | Galla,Arab;Abergelle,Gumuz | -0.000220737 | 3.48E-05 | -6.3382 |
| 42 | Galla,Gumuz;Abergelle,Arab | 0.000730427 | 3.35E-05 | 21.8029 |
| 43 | Galla,Abergelle;Gumuz,Guera | 0.000275692 | 3.99E-05 | 6.90366 |
| 44 | Galla,Guera;Abergelle,Gumuz | -0.000476888 | 3.79E-05 | -12.5916 |
| 45 | Galla,Gumuz;Abergelle,Guera | -0.000201197 | 3.96E-05 | -5.07771 |
| 46 | Galla,Abergelle;Gumuz,UknMorocco | 0.000453102 | 3.77E-05 | 12.0203 |
| 47 | Galla,Gumuz;Abergelle,UknMorocco | -0.000327468 | 3.54E-05 | -9.2565 |
| 48 | Galla,UknMorocco;Abergelle,Gumuz | -0.000780569 | 3.49E-05 | -22.3717 |
| 49 | Galla,Abergelle;Gumuz,Woyto-Guji | -0.00102853 | 3.22E-05 | -31.9014 |
| 50 | Galla,Gumuz;Abergelle,Woyto-Guji | -0.000416378 | 3.38E-05 | -12.3062 |
| 51 | Galla,Woyto-Guji;Abergelle,Gumuz | 0.000612147 | 2.94E-05 | 20.8166 |
| 52 | Galla,Abergelle;Gumuz,Oromo | 0.00118305 | 3.22E-05 | 36.791 |
| 53 | Galla,Gumuz;Abergelle,Oromo | 0.000811348 | 3.18E-05 | 25.5374 |
| 54 | Galla,Oromo;Abergelle,Gumuz | -0.000371701 | 3.44E-05 | -10.8058 |
