# Supplementary material for: Whole-genome resequencing reveals genomic variation and dynamics in Ethiopian indigenous goats
Source: Front Genet. 2024 May 24;15:1353026. doi: 10.3389/fgene.2024.1353026 (PMC11156998; doi:10.3389/fgene.2024.1353026)
Supplement: Supplementary file 9 [file Table3.DOCX]

**Supplementary Table S3:** InDel statistics for each goat population

| **Population** | **Total number of InDels** | **Average per sample** | **Number of Insertions** | **Number of deletions** | **Reported InDels (rs)** | **Novel InDels** | **dbSNP (%)** | | **transition-to-transversion (*ts/tv*) ratio** |
| --- | --- | --- | --- | --- | --- | --- | --- | --- | --- |
|  |  |  |  |  |  |  | **rs** | **novel** |  |
| ARB | 1,823,938 | 862,959 | 828,360 | 995,578 | 353,604 | 1,470,334 | 19.39 | 80.61 | 2.30 |
| FEL | 1,911,951 | 835,348 | 857,076 | 1,054,875 | 383,715 | 1,528,236 | 20.07 | 79.93 | 2.30 |
| ORO | 1,824,730 | 847,620 | 825,643 | 999,087 | 351,739 | 1,472,991 | 19.28 | 80.72 | 2.30 |
| ABR | 1,929,864 | 976,619 | 896,970 | 1,032,894 | 351,515 | 1,578,349 | 18.21 | 81.79 | 2.27 |
| KEF | 1,910,158 | 660,074 | 872,679 | 1,037,479 | 372,662 | 1,537,496 | 19.51 | 80.49 | 2.25 |
| GUM | 1,739,360 | 962,116 | 797,045 | 942,315 | 319,335 | 1,420,025 | 18.36 | 81.64 | 2.29 |
| WGU | 1,953,928 | 965,809 | 887,880 | 1,066,048 | 388,506 | 1,565,422 | 19.88 | 80.11 | 2.31 |
| GAL | 1,973,805 | 953,,145 | 896,188 | 1,077,617 | 391,179 | 1,582,626 | 19.82 | 80.18 | 2.29 |
| UNK | 2,033,758 | 904,149 | 900,464 | 1,133,294 | 447,785 | 1,585,973 | 22.02 | 77.98 | 2.29 |
| THY | 1,757,884 | 938,703 | 816,560 | 941,324 | 318,880 | 1,439,004 | 18.14 | 81.86 | 2.27 |
| GUE | 1,851,019 | 884,809 | 854,534 | 996,485 | 355,442 | 1,495,577 | 19.20 | 80.80 | 2.29 |
| SAN | 1,768,578 | 797,437 | 846,837 | 921,741 | 310,913 | 1,457,665 | 17.58 | 82.42 | 2.26 |
| TIB | 1,895,948 | 825,717 | 837,036 | 1,058,912 | 343,528 | 1,552,420 | 18.12 | 81.88 | 2.30 |
