# Supplementary material for: Whole-genome resequencing reveals genomic variation and dynamics in Ethiopian indigenous goats
Source: Front Genet. 2024 May 24;15:1353026. doi: 10.3389/fgene.2024.1353026 (PMC11156998; doi:10.3389/fgene.2024.1353026)
Supplement: Supplementary file 10 [file Table4.DOCX]

**Supplementary Table S4:** Results of *f3* statistics for the study populations as generated with TreeMix

| **S. No** | **Breeds (A; B, C)** | **f3-statistics** | **Standard Error** | **Z** |
| --- | --- | --- | --- | --- |
| 1 | Abergelle;Boran,Saanen | 0.0030465 | 4.55E-05 | 66.9278 |
| 2 | Boran;Abergelle,Saanen | 0.00234849 | 4.48E-05 | 52.3955 |
| 3 | Saanen;Boran,Abergelle | 0.0268598 | 9.17E-05 | 292.889 |
| 4 | Abergelle;Boran,Gumuz | 0.0038312 | 3.79E-05 | 101.115 |
| 5 | Boran;Abergelle,Gumuz | 0.00156379 | 3.73E-05 | 41.9264 |
| 6 | Gumuz;Boran,Abergelle | 0.00339484 | 4.10E-05 | 82.812 |
| 7 | Abergelle;Boran,Keffa | 0.00217472 | 3.35E-05 | 64.9655 |
| 8 | Boran;Abergelle,Keffa | 0.00322027 | 3.82E-05 | 84.2633 |
| 9 | Keffa;Boran,Abergelle | 0.00357851 | 3.32E-05 | 107.89 |
| 10 | Abergelle;Boran,Thyolo | 0.00435251 | 4.51E-05 | 96.4205 |
| 11 | Boran;Abergelle,Thyolo | 0.00104248 | 4.25E-05 | 24.5233 |
| 12 | Thyolo;Boran,Abergelle | 0.0135241 | 7.08E-05 | 191.047 |
| 13 | Abergelle;Boran,Fellata | 0.00339792 | 3.78E-05 | 89.9906 |
| 14 | Fellata;Boran,Abergelle | 0.00348461 | 3.74E-05 | 93.2082 |
| 15 | Boran;Abergelle,Fellata | 0.00199707 | 3.46E-05 | 57.7932 |
| 16 | Abergelle;Boran,Arab | 0.00288004 | 3.70E-05 | 77.7646 |
| 17 | Arab;Boran,Abergelle | 0.00467454 | 4.27E-05 | 109.425 |
| 18 | Boran;Abergelle,Arab | 0.00251496 | 3.78E-05 | 66.5967 |
| 19 | Abergelle;Boran,Guera | 0.00355551 | 4.30E-05 | 82.6163 |
| 20 | Boran;Abergelle,Guera | 0.00183948 | 3.94E-05 | 46.6583 |
| 21 | Guera;Boran,Abergelle | 0.0116452 | 5.92E-05 | 196.819 |
| 22 | Abergelle;Boran,UknMorocco | 0.0033781 | 3.95E-05 | 85.5861 |
| 23 | Boran;Abergelle,UknMorocco | 0.00201689 | 3.74E-05 | 53.9778 |
| 24 | UknMorocco;Boran,Abergelle | 0.00714501 | 4.70E-05 | 152.062 |
| 25 | Abergelle;Boran,Woyto-Guji | 0.00485972 | 3.93E-05 | 123.749 |
| 26 | Boran;Abergelle,Woyto-Guji | 0.000535268 | 3.12E-05 | 17.1604 |
| 27 | Woyto-Guji;Boran,Abergelle | 0.000845518 | 3.24E-05 | 26.0571 |
| 28 | Abergelle;Boran,Oromo | 0.00264815 | 3.51E-05 | 75.4888 |
| 29 | Boran;Abergelle,Oromo | 0.00274684 | 3.78E-05 | 72.5728 |
| 30 | Oromo;Boran,Abergelle | 0.00398283 | 3.76E-05 | 105.981 |
| 31 | Abergelle;Boran,Tibetan | 0.00309189 | 4.37E-05 | 70.6979 |
| 32 | Boran;Abergelle,Tibetan | 0.0023031 | 4.32E-05 | 53.3556 |
| 33 | Tibetan;Boran,Abergelle | 0.0209418 | 7.34E-05 | 285.121 |
| 34 | Boran;Saanen,Gumuz | 0.00121444 | 4.72E-05 | 25.755 |
| 35 | Gumuz;Boran,Saanen | 0.00374419 | 4.87E-05 | 76.9585 |
| 36 | Saanen;Boran,Gumuz | 0.0279938 | 9.42E-05 | 297.182 |
| 37 | Boran;Saanen,Keffa | 0.00336026 | 4.74E-05 | 70.9184 |
| 38 | Keffa;Boran,Saanen | 0.00343852 | 4.40E-05 | 78.163 |
| 39 | Saanen;Boran,Keffa | 0.025848 | 8.98E-05 | 287.944 |
| 40 | Boran;Saanen,Thyolo | 0.00216507 | 5.56E-05 | 38.9107 |
| 41 | Saanen;Boran,Thyolo | 0.0270432 | 9.78E-05 | 276.491 |
| 42 | Thyolo;Boran,Saanen | 0.0124015 | 7.43E-05 | 166.906 |
